# A One-Step Microwave-Assisted Synthetic Method for an O/S-Chemoselective Route to Derivatives of the First Adenosine A3 PET Radiotracer

**DOI:** 10.3390/molecules19044076

**Published:** 2014-04-02

**Authors:** Karem Shanab, Catharina Neudorfer, Wolfgang Holzer, Markus Mitterhauser, Wolfgang Wadsak, Helmut Spreitzer

**Affiliations:** 1 Division of Nuclear Medicine, Department of Biomedical Imaging and Image-guided Therapy, Medical University of Vienna, Waehringer Guertel 18-20, 1090 Vienna, Austria; 2 Department of Pharmaceutical Chemistry, University of Vienna, Althanstrasse 14, 1090 Vienna, Austria

**Keywords:** chemoselective, alkylation, microwave, adenosine A3, PET, radiotracer

## Abstract

The synthesis of reference standards and expected *in vivo* metabolites of the first adenosine A3 PET radiotracer [^18^F]FE@SUPPY [^18^F]fluoroethyl 4,6-diethyl-5-[(ethyl-sulfanyl)carbonyl]-2-phenylpyridine-3-carboxylate) was achieved by using a straightforward microwave assisted alkylation method, which allowed O/S-chemoselective alkylation of the starting material **1** to give each target compound 2–8 in a single step.

## 1. Introduction

**Figure 1:**
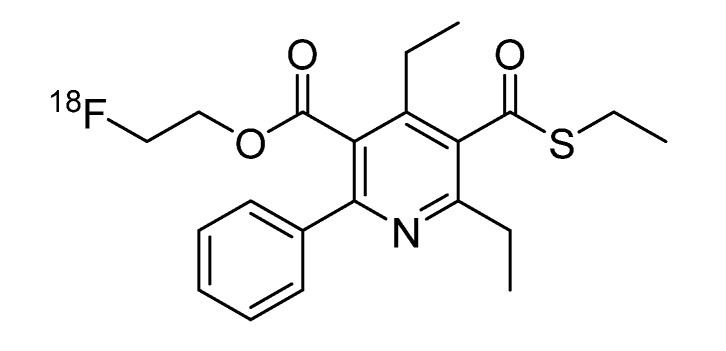
[^18^F]FE@SUPPY.

Adenosine plays a pivotal role as a key modulator in the human body and acts through four different receptor subtypes: A_1 _(A1AR), A_2A_ (A2AAR), A_2B_ (A2BAR) and A_3_ (A3AR) receptors [1]. The A3AR is known to be involved in a variety of diseases, such as asthma, neurodegenerative disorders, inflammatory diseases and lately also showed to be highly expressed on the surface of various tumor cells [2,3]. There exists much information on the distribution of the A_1_ and A_2A_ receptors because good pharmacological tools—including radioligands—are available. In the case of A_3_ receptors, these tools were not yet available, and thus, there were still many open questions regarding the distribution and involvement in pathophysiological processes. The fluoroethylester FE@SUPPY (5-(2-fluoroethyl)2,4-diethyl-3-(ethylsulfanyl-carbonyl)-6-phenylpyridine-5-carboxylate), which was evaluated by Li *et al* [4], displays high affinity as well as high selectivity for the A3AR. These excellent features attested FE@SUPPY as an outstanding ligand for the A3AR. Based on this preliminary work, we recently introduced the first potential PET (positron emission tomography) tracer [^18^F]FE@SUPPY for the A3AR (Figure 1) [5].

PET excels as a suitable technique for the collection of the lacking data in the living organism and at the same time enables direct receptor quantification of receptor densities. As a prerequisite for successful PET imaging, suitable radioligands with high affinity, high selectivity and low unspecific binding are required [6]. For quality control of these radioligands, non-radioactive reference compounds needed to be prepared. These reference compounds did not only serve as reference standards for the radiometabolites, but were as well employed in preclinical pharmacokinetic experiments of the PET radiotracer [^18^F]FE@SUPPY [7]. A one-step approach for the preparation of the reference substances was sought in order to promptly achieve those derivatives. In this paper we present a straightforward, O/S-chemoselective and microwave assisted alkylation method for the synthesis of several reference compounds of FE@SUPPY-derived A3AR radiotracers starting from the universal precursor SUPPY: 0 (**1**).

## 2. Results and Discussion

**Scheme 1:**
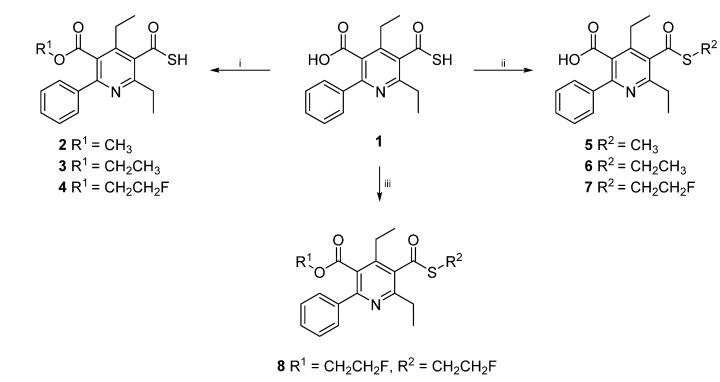
One step O/S-chemoselective alkylation of SUPPY:0 (**1**).

4,6-Diethyl-2-phenyl-5-(sulfanylcarbonyl)pyridine-3-carboxylic acid) (SUPPY:0, **1**) was prepared according to our previously published method [8]. Chemoselective alkylation of the carboxylic acid moiety of **1** to afford compounds **2**–**4** was achieved under microwave assisted reaction conditions in acetonitrile in 10–15 min at 150 °C (300–350 W). Methyl trifluoromethanesulfonate, ethyl trifluoromethanesulfonate, or 2-fluoroethyl trifluoromethanesulfonate, respectively, were used as alkylating agents with cesium carbonate as base and an ionic additive to improve the microwave heating of the solvent. NMR analysis of the crude product disclosed a mean oxygen/sulfur alkylation ratio of 5:1 for the above described method. Alternatively, methylation of **1** to give product **2** was accomplished chemoselectively by treatment with diazomethane in toluene/methanol at room temperature in 30 min. To obtain the *S*-alkylated products **5**–**7**, SUPPY:0 (**1**) was treated with either methyl trifluoromethanesulfonate, ethyl trifluoromethanesulfonate, or 2-fluoroethyl trifluoromethanesulfonate under microwave assisted reaction conditions 10 min at 150 °C (300–350 W). The addition of ten equivalents of sodium iodide and DMF as solvent were discovered to be the crucial modifications to achieve the chemoselective reaction at the sulfur atom to afford compounds **5**–**7** with a mean sulfur/oxygen alkylation ratio of 4:1 (NMR analysis of the crude product). Interestingly, compound **8** could be made accessible in one single step from SUPPY:0 (**1**) and 2.4 equivalents of 2-fluoroethyl trifluoromethanesulfonate by increasing microwave power to 600 W (170 °C, 5 min) (Scheme 1).

*Reagents and Conditions*: (i) Cs_2_CO_3_, CH_3_CN, methyl trifluoromethanesulfonate, ethyl trifluoromethanesulfonate, or 2-fluoroethyl trifluoromethanesulfonate, 10–15 min, 150 °C, 300 W; (ii) NaI, DMF, methyl trifluoromethanesulfonate, ethyl trifluoromethanesulfonate, or 2-fluoroethyl trifluoromethanesulfonate, 10 min, 100–150 °C, 300 W; (iii): NaI, DMF, 2-fluoroethyl trifluoromethanesulfonate, 5 min, 170 °C, 600 W.

## 3. Experimental

### 3.1. General Information

The NMR spectra were recorded with a Bruker DPX 200 spectrometer (200 MHz for ^1^H, 50 MHz for ^13^C) or a Bruker Avance 500 spectrometer (500 MHz for ^1^H, 125 MHz for ^13^C, 50 MHz for ^15^N, 470 MHz for ^19^F) at 297 K using “directly” detecting broadband observe (BBFO) probes. The center of the solvent (residual) signal was used as an internal standard which was related to TMS with δ 7.26 ppm (^1^H in CDCl_3_), δ 2.49 ppm (^1^H in DMSO-*d*_6_), δ 77.0 ppm (^13^C in CDCl_3_), and δ 39.50 ppm (^13^C in DMSO-*d*_6_), ^19^F NMR spectra were referenced via *Ξ* ratio. Coupling constants (*J*) are quoted in Hz. The following abbreviations were used to show the multiplicities: s: singlet, d: doublet, t: triplet, q: quadruplet, dd: doublet of doublet, m: multiplet. Infrared spectra (IR) were recorded on a Perkin Elmer FT-IR spectrophotometer (model spectrum 1000). Mass spectra (MS) were recorded on a Shimadzu GC/MS-combined GC/MS-Q95050A GC-17A instrument. High resolution mass spectra (HRMS) were recorded under electrospray ionization (ESI), using a Finnigan MAT 900 S instrument. High-resolution mass spectrometry was carried out on an electrospray ionization mass spectrometer with a micro-TOF analyzer. CHN-analysis was performed by the microanalytical laboratory of the University of Vienna. Melting points were determined through a Reichert-Kofler hot-stage microscope. Thin-layer chromatography analyses were performed using precoated silica gel plates (Merck, Darmstadt, Germany). Column chromatography was performed with silica gel 60 or RP-18 silica gel, respectively (Merck). Microwave experiments were carried out in a Synthos 3000 microwave oven (Anton Paar, SXQ80 rotor, Graz, Austria) with an internal temperature probe.

### 3.2. General Procedure for the Synthesis of Compounds **2**–**4**

4,6-Diethyl-2-phenyl-5-(sulfanylcarbonyl)pyridine-3-carboxylic acid (0.10 g, 0.32 mmol), Cs_2_CO_3_ (0.22 g, 0.66 mmol) and the according trifluoromethanesulfonate (0.72 mmol) were suspended in CH_3_CN (10 mL). The reaction mixture was heated in the microwave oven to 150 °C at 300 W for 10 min (**3**, **4**) or 15 min (**2**), respectively. Then the solvent was removed under reduced pressure and the product purified by column chromatography.

*2,4-Diethyl-5-(methoxycarbonyl)-6-phenylpyridine-3-carbothioic S-acid* (**2**). Purification: RP-18 silica gel, CH_3_CN/H_2_O 9/1. Yield: 30 mg (29%), white crystals, m.p. 161–162 °C; ^1^H-NMR (200 MHz, CDCl_3_) δ (ppm): 7.66 (q, 2H, *J* = 3.9 Hz), 7.45 (t, 3H, *J* = 3.42 Hz), 3.66 (s, 3H), 3.06 (t, 2H, *J* = 7.46 Hz), 2.92 (m, 2H), 1.40 (t, 3H, *J* = 7.44 Hz), 1.28 (t, 3H, *J* = 7.32 Hz); ^13^C-NMR (50 MHz, CDCl_3_) δ (ppm): 189.4, 168.6, 160.1, 157.9, 149.2, 139.3, 130.5, 129.2, 128.4, 128.2, 126.5, 52.4, 29.4, 15.8, 14.1; IR (KBr): *v* (cm^−1^) 3427, 2974, 2940, 2877, 1728, 1559, 1439, 1403; MS: *m/z* (%): 328 (M^+^, 1.29), 327 (5), 297 (21), 296 (100), 264 (5), 236 (10), 208 (5), 77 (5); HRMS: *m/z* calculated for C_18_H_18_NO_3_S (M^+^–SH): 296.1287. Found: 296.1289; CHN: Anal. calculated for C_18_H_19_NO_3_S: C, 65.63; H, 5.81; N, 4.25. Found: C, 65.37; H, 5.50; N, 4.41.

*5-(Ethoxycarbonyl)-2,4-diethyl-6-phenylpyridine-3-carbothioic S-acid* (**3**). Purification: RP-18 silica gel, CH_3_CN/H_2_O 9/1. Yield: 120 mg (50%), yellow oil; ^1^H-NMR (200 MHz, CDCl_3_): δ (ppm) 7.63 (m, 2H), 7.44 (m, 3H), 4.14 (q, 2H, *J* = 7.20 Hz), 3.05 (q, 2H, *J* = 7.44 Hz), 2.93 (q, 2H, *J* = 7.44 Hz), 1.40 (t, 3H, *J* = 7.32 Hz), 1.29 (t, 3H, *J* = 7.46 Hz), 1.03 (t, 3H, *J* = 7.20 Hz); ^13^C-NMR (50 MHz, CDCl_3_): δ (ppm) 189.3, 167.9, 160.0, 158.0, 149.5, 139.1, 130.6, 129.2, 128.4, 127.0, 61.8, 29.3, 24.4, 15.9, 14.3, 13.6; IR (KBr): ν (cm^−1^) 3419, 3058, 2971, 2939, 2875, 1724, 1559, 1475, 1458, 1445, 1403, 1374, 1344, 1282, 1250, 1215, 1170, 1145, 1092, 1077, 1016, 963; MS: *m/z* (%) 344 (2), 343 (3), 341 (43), 312 (19), 311 (17), 310 (100), 282 (29), 254 (13), 236 (11), 77 (14); HRMS: *m/z* calculated for C_19_H_21_NO_3_S: 343.1242. Found: 343.1235.

*2,4-Diethyl-5-[(2-fluoroethoxy)carbonyl]-6-phenylpyridine-3-carbothioic S-acid* (**4**). Purification: RP-18 silica gel, CH_3_CN. Yield: 113 mg (95%), colorless crystals, m.p. 45–47 °C; ^1^H-NMR (300 MHz, CDCl_3_): δ (ppm) 7.64 (m, 2H, Ph H-2/H-6), 7.46 (m, 3H, Ph H-3/H-4/H-5), 4.39/4.29 (m, 2H, FCH_2_), 4.34/4.29 (m, 2H, CH_2_O), 3.05 (q, 2H, *J* = 7.50 Hz, C-2CH_2_), 2.93 (q, 2H, *J* = 7.50 Hz, C-4CH_2_) 1.40 (t, 3H, *J* = 7.50 Hz, C-2CH_2_CH_3_), 1.29 (t, 3H, *J* = 7.50 Hz, C-4CH_2_CH_3_); ^13^C-NMR (75 MHz, CDCl_3_): δ (ppm) 189.3 (COSH), 167.9 (COO), 160.4 (C-2), 158.3 (C-6), 149.5 (C-4), 139.3 (Ph C-1), 130.6 (C-3), 129.3 (Ph C-4), 128.5 (Ph C-3/C-5), 128.3 (Ph C-2/ C-6), 126.2 (C-5), 80.5 (d, FCH_2_, ^1^*J*_CF_ = 172.0 Hz), 64.5 (d, CH_2_O ^2^*J*_CF_ = 20.2 Hz), 29.5 (C-2CH_2_), 24.4 (C-4CH_2_), 15.9 (C-4CH_2_CH_3_), 14.3 (C-2CH_2_CH_3_); IR (KBr): ν (cm^−1^) 3439, 2975, 2938, 2877, 1731, 1556, 1495, 1448, 1403, 1377, 1278, 1250, 1166, 1144, 1077, 1060, 1033, 960; MS: *m/z* (%) 362 (1), 361 (3), 359 (31), 329 (21), 328 (100), 312 (14), 254 (15), 236 (12), 77 (20), 47 (48); CHN: Anal. calculated for C_19_H_20_FNO_3_S∙0.2 H_2_O: C, 62.52; H, 5.63; N, 3.84. Found: C, 62.45; H, 5.44; N, 3.73.

### 3.3. Alternative Method for the Synthesis of 2,4-Diethyl-5-(methoxycarbonyl)-6-phenylpyridine-3-carbothioic S-acid (**2**)

To a stirred solution of 4,6-diethyl-2-phenyl-5-(sulfanylcarbonyl)pyridine-3-carboxylic acid (0.20 g, 0.63 mmol) in toluene/MeOH (9/6), trimethylsilyldiazomethane (0.4 mL, 0.63 mmol) was added dropwise until the yellow color of the mixture persisted. Thereafter, the solution was stirred for another 30 min at room temperature. The solvent was removed by reduced pressure and product **2** purified by reversed phase column chromatography (RP-18 silica gel, CH_3_CN/H_2_O 9/1). Yield: 141 mg (68%).

### 3.4. General Procedure for the Synthesis of Compounds **5**‒**7**

4,6-Diethyl-2-phenyl-5-(sulfanylcarbonyl)pyridine-3-carboxylic acid (0.10 g, 0.32 mmol), NaI (0.48 g, 3.17 mmol) and the according trifluoromethanesulfonate (0.72 mmol) were dissolved in DMF (10 mL). The reaction mixture was heated in the microwave oven at 300 W and 100 °C for 10 min (**6**, **7**) or 15 min (**5**), respectively. The solvent was removed under reduced pressure and the crude product purified by column chromatography.

*4,6-Diethyl-5-[(methylsulfanyl)carbonyl]-2-phenylpyridine-3-carboxylic acid* (**5**). Purification: silica gel 60, petrol ether/EtOAc 8/2. Yield: 52 mg (49%), colorless crystals, m.p. 243–244 °C; ^1^H-NMR (200 MHz, DMSO-*d*_6_) δ (ppm): 13.63 (s, 1H), 7.55 (q, 5H, *J* = 3.54 Hz), 2.69 (m, 7H), 1.19 (m, 6H); ^13^C-NMR (50 MHz, DMSO-*d*_6_) δ (ppm): 195.3, 169.3, 157.8, 155.0, 146.4, 139.1, 132.7, 129.0, 128.3, 127.9, 28.3, 23.8, 15.6, 13.8, 12.4; IR (KBr): *v* (cm^−1^) 3441, 2983, 2938, 2868, 2492, 1921, 1712, 1667, 1554; MS: *m/z* (%): 330 (M^+^, 0.12), 298 (1), 283 (17), 282 (100), 236 (6), 180 (5), 127 (7), 77 (10), 47 (10), 45 (6); HRMS: *m/z* calculated for C_18_H_19_NO_3_S: 329.1086. Found: 329.1076; CHN: Anal. calculated for C_18_H_19_NO_3_S: C, 65.63; H, 5.81; N, 4.25. Found: C, 65.64; H, 5.81; N, 4.06.

*4,6-Diethyl-5-[(ethylsulfanyl)carbonyl]-2-phenylpyridine-3-carboxylic acid* (**6**). Purification: silica gel 60, petrol ether/EtOAc 8/2.Yield: 94 mg (85%), colorless crystals, m.p. 200–202 °C; ^1^H-NMR (300 MHz, DMSO-* d*_6_): δ (ppm) 13.20 (br s, 1H), 7.65 (m, 2H), 7.45 (m, 3H), 3.12 (q, 2H, *J* = 7.30 Hz), 2.76 (q, 2H, *J *= 7.50 Hz), 2.66 (q, 2H, *J* = 7.50 Hz), 1.32 (t, 3H, *J* = 7.50 Hz), 1.24 (t, 3H, *J* = 7.50 Hz), 1.17 (t, 3H, *J* = 7.50 Hz); ^13^C-NMR (75 MHz, DMSO-*d*_6_): δ (ppm) 194.8, 169.2, 155.0, 146.2, 139.1, 132.6, 132.6, 128.3, 128.2, 127.9, 28.2, 24.2, 23.6, 15.5, 14.4, 13.7; IR (KBr): *v* (cm^−1^) 3443, 2977, 2491, 1925, 1711, 1661, 1555, 1453, 1413, 1376, 1260, 1184, 1149, 1078; MS: *m/z* (%) 344 (1), 343 (1), 327 (1), 326 (4), 283 (18), 282 (100), 236 (5), 180 (5, 126 (7), 91 (7), 77 (11); CHN: Anal. calculated for C_19_H_21_NO_3_S: C, 66.45; H, 6.16; N, 4.08. Found: C, 66.18; H, 6.21; N, 3.96.

*4,6-Diethyl-5-{[(2-fluoroethyl)sulfanyl]carbonyl}-2-phenylpyridine-3-carboxylic acid* (**7**). Purification: RP-18 silica gel, CH_3_CN/H_2_O 9/1. Yield: 30 mg (52%), colorless crystals, m.p. 195–196 °C; ^1^H-NMR (200 MHz, DMSO-*d*_6_): δ (ppm) 13.67 (s, 1H), 7.65 (m, 2H), 7.46 (t, 3H, *J* = 3.42 Hz), 4.79 (t, 1H, *J* = 5.68 Hz), 4.55 (t, 1H, *J* = 5.68 Hz), 3.58 (t, 1H, *J* = 5.68 Hz), 3.46 (t, 1H, *J* = 5.54 Hz), 2.71 (m, 4H), 1.20 (m, 6H); ^13^C-NMR (50 MHz, DMSO-*d*_6_): δ (ppm) 194.2, 169.2, 157.7, 155.2, 146.4, 139.1, 132.4, 129.0, 128.3, 128.0, 81.6 (d, ^1^*J*_CF_ = 166.5 Hz), 29.9 (d, ^2^*J*_CF_ = 20.7 Hz), 28.3, 23.7, 15.6, 13.8; IR (KBr): *v* (cm^−1^) 3446, 2974, 2454, 1966, 1708, 1671, 1555, 1412, 1287, 1183; MS: *m/z* (%) 283 (16), 282 (100), 77 (29), 69 (14), 59 (20), 45 (15), 43 (20), 41 (16); CHN: Anal. calculated for C_19_H_20_FNO_3_S: C, 63.14; H, 5.58; N, 3.88. Found: C, 63.04; H, 5.54; N, 3.73.

### 3.5. Synthesis of 2-Fluoroethyl 4,6-Diethyl-5-{[(2-fluoroethyl)sulfanyl]carbonyl}-2-phenylpyridine-3-carboxylate (**8**)

4,6-Diethyl-2-phenyl-5-(sulfanylcarbonyl)pyridine-3-carboxylic acid (0.073 g, 0.23 mmol), NaI (0.348 g, 2.3 mmol) and 2-fluoroethyl trifluoromethanesulfonate (0.11 g, 0.56 mmol) were dissolved in DMF (10 mL). The mixture was heated in the microwave oven at 600 W and 170 °C for 5 min. The solvent was then removed under reduced pressure and the crude product purified by RP-18 column chromatography (CH_3_CN/H_2_O 9/1). Yield: 67 mg (72%), orange oil; ^1^H-NMR (200 MHz, CDCl_3_) δ (ppm): 7.60 (m, 2H), 7.43 (m, 3H), 4.77 (t, 1H, *J = *5.99 Hz), 4.54 (t, 1H, *J = *5.94 Hz), 4.42 (m, 1H), 4.34 (q, 1H, *J = *2.48 Hz), 4.18 (d, 2H, *J = *5.18 Hz), 3.52 (t, 1H, *J = *5.99 Hz), 3.41 (t, 1H, *J = *5.94 Hz), 2.87 (q, 2H, J= 7.49 Hz), 2.73 (q, 2H, *J =* 7.53 Hz), 1.34 (t, 3H, *J =* 7.51 Hz), 1.24 (t, 3H, *J =* 7.57 Hz); ^13^C-NMR (50 MHz, CDCl_3_) δ (ppm): 194.3, 168.1, 159.6, 157.5, 148.2, 139.6, 132.6, 129.0, 128.4, 128.3, 126.0, 81.3 (d, FCH_2_CH_2_O, ^1^*J*_CF_ = 170.5 Hz), 80.5 (d, FCH_2_CH_2_S, ^1^*J*_CF_ = 170.2 Hz), 64.1 (d, CH_2_O ^2^*J*_CF_ = 20.3 Hz), 30.0 (d, CH_2_S ^2^*J*_CF_ = 22.2 Hz), 29.2, 24.2, 15.7, 14.0; IR (KBr): *v* (cm^−1^) 3447, 2977, 2879, 1734, 1675, 1558, 1496; MS: *m/z* (%): 407 (M^+^, 0.59), 329 (21), 328 (100), 282 (6), 254 (6), 236 (5), 47 (6); HRMS: m/z calculated for C_21_H_24_O_3_F_2_NS: 408.1445. Found: 408.1450.

## 4. Conclusions

In this paper, a straightforward and microwave assisted alkylation method for seven different compounds **2**‒**8** was presented, starting from the universal precursor SUPPY:0 (**1**). O/S chemoselectivity could be achieved through variation of the additive and the solvent. Compounds **2**–**4** could be made accessible through the utilization of Cs_2_CO_3_, whereas NaI addition proved to be essential for *S*-chemoselective alkylation and therefore, for the preparation of substances **5**–**7**. However, for the synthesis of compound **8**, NaI and an increase in microwave power provided the required reaction conditions to achieve conversion. All compounds **2**‒**8** were subjected to detailed NMR spectroscopic studies. These target structures served as reference standards in preclinical pharmacokinetic experiments of SUPPY:0 derived radiotracers.

## Supplementary Materials

^1^H and ^13^C-NMR spectra, IR spectra, mass spectra and HRMS spectra are available at: http://www.mdpi.com/1420-3049/19/4/4076/s1.
