# Supplementary material for: A One-Step Microwave-Assisted Synthetic Method for an O/S-Chemoselective Route to Derivatives of the First Adenosine A3 PET Radiotracer
Source: Molecules. 2014 Apr 2;19(4):4076–82. doi: 10.3390/molecules19044076 (PMC6271051; doi:10.3390/molecules19044076)

Supplementary File

Figure S1. ^{1}H-NMR spectrum compound **2**.

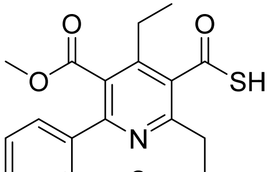
2

vscosh 2,4-diethyl-5-(methoxycarbonyl)-6-phenylpyridine-3-carbothioic S-acid in CDC.

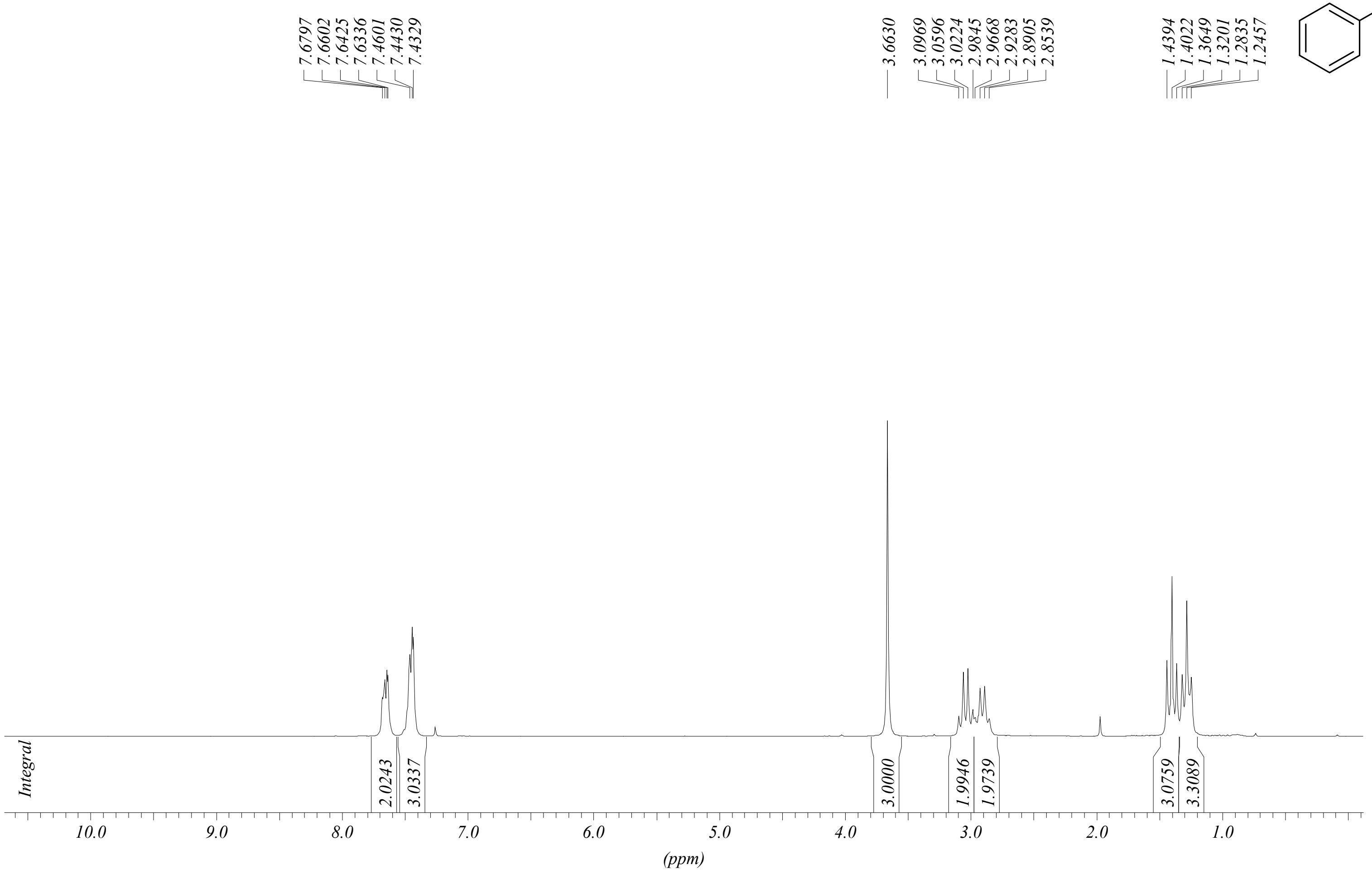

Figure S2. ^{13}C-NMR spectrum compound **2**.

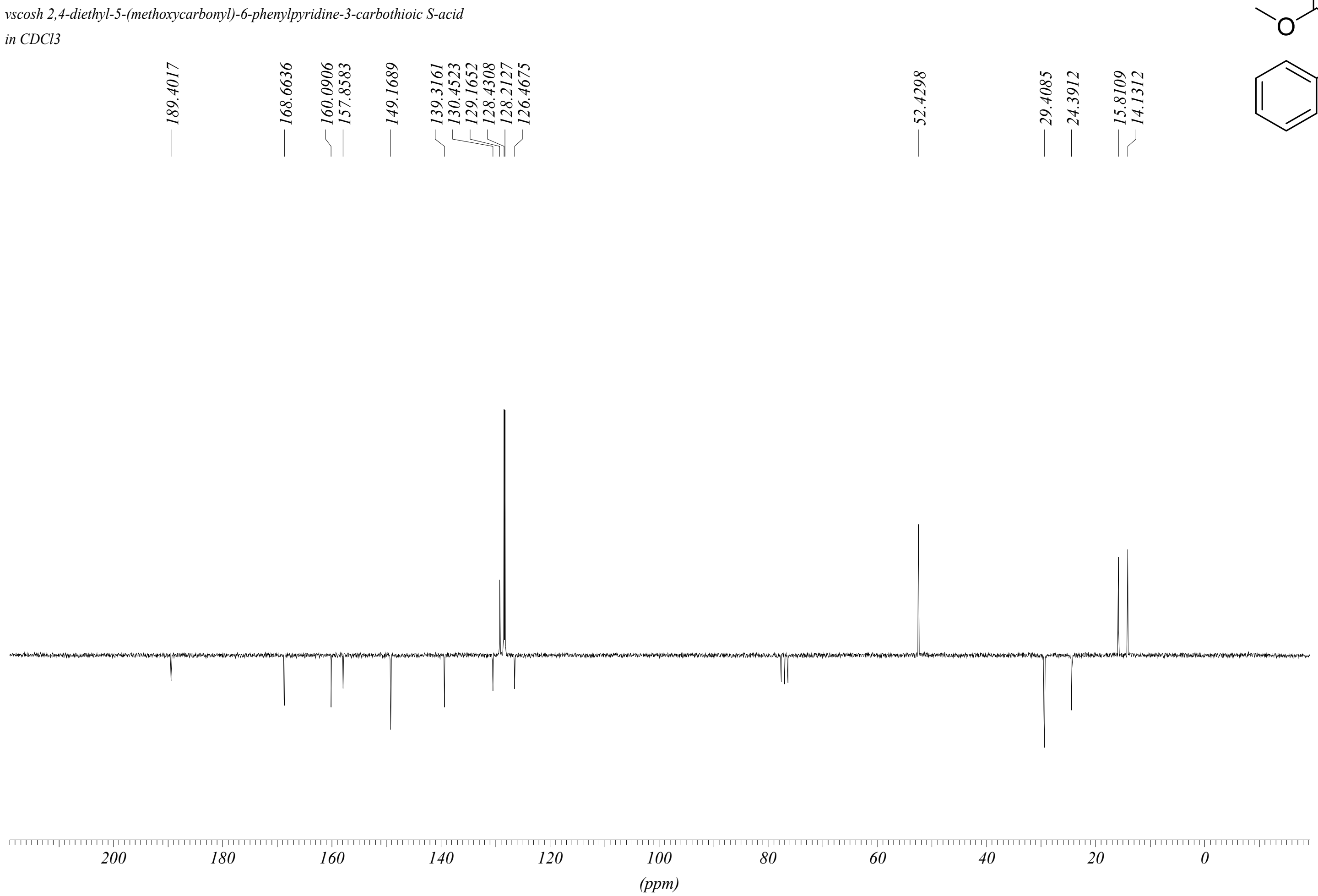

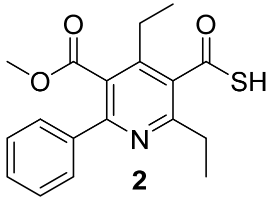

2

Figure S3. IR spectrum compound **2**.

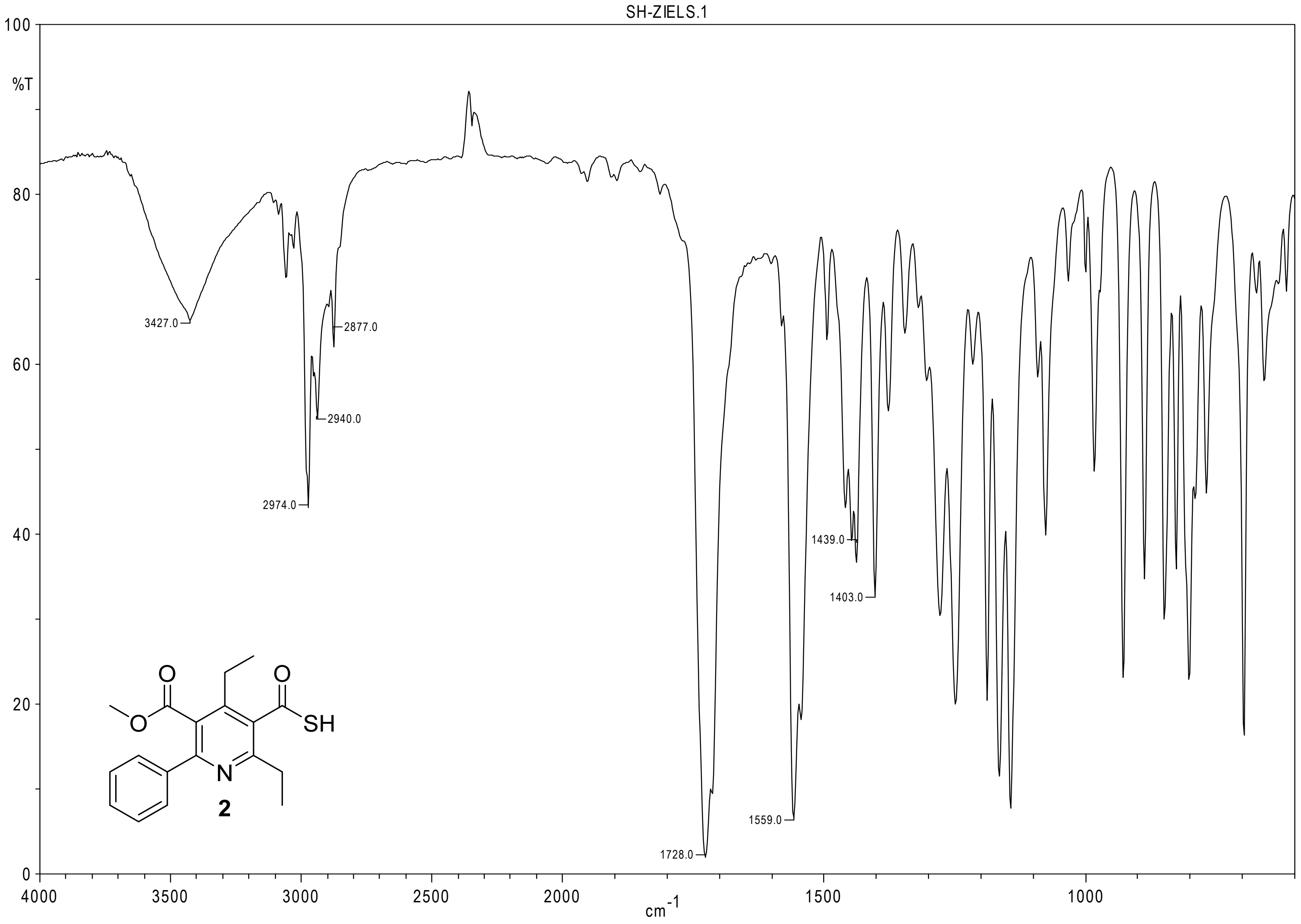

Figure S4. Mass spectrum compound **2**.

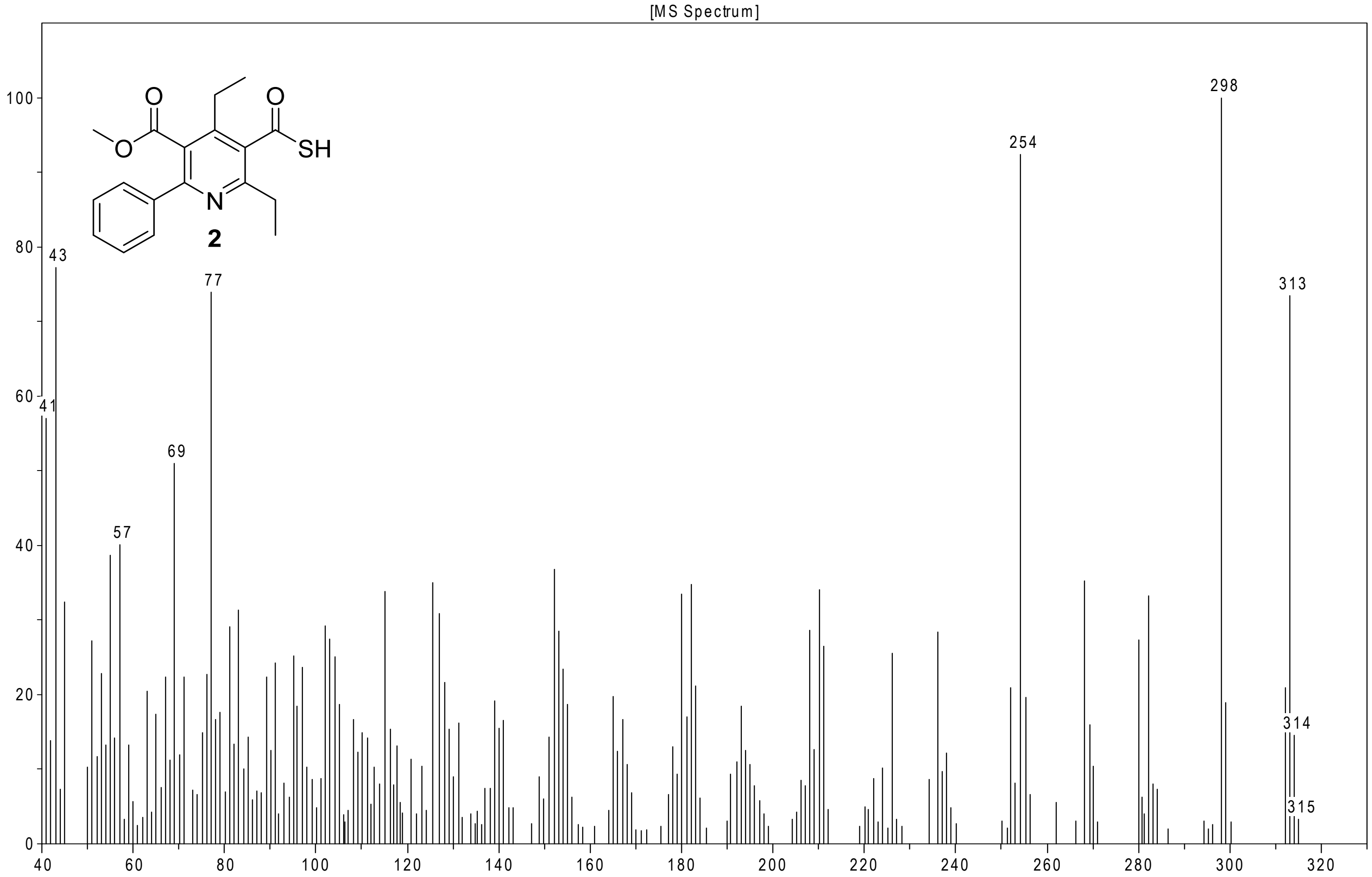

Figure S5. ^{1}H-NMR spectrum compound **3**.

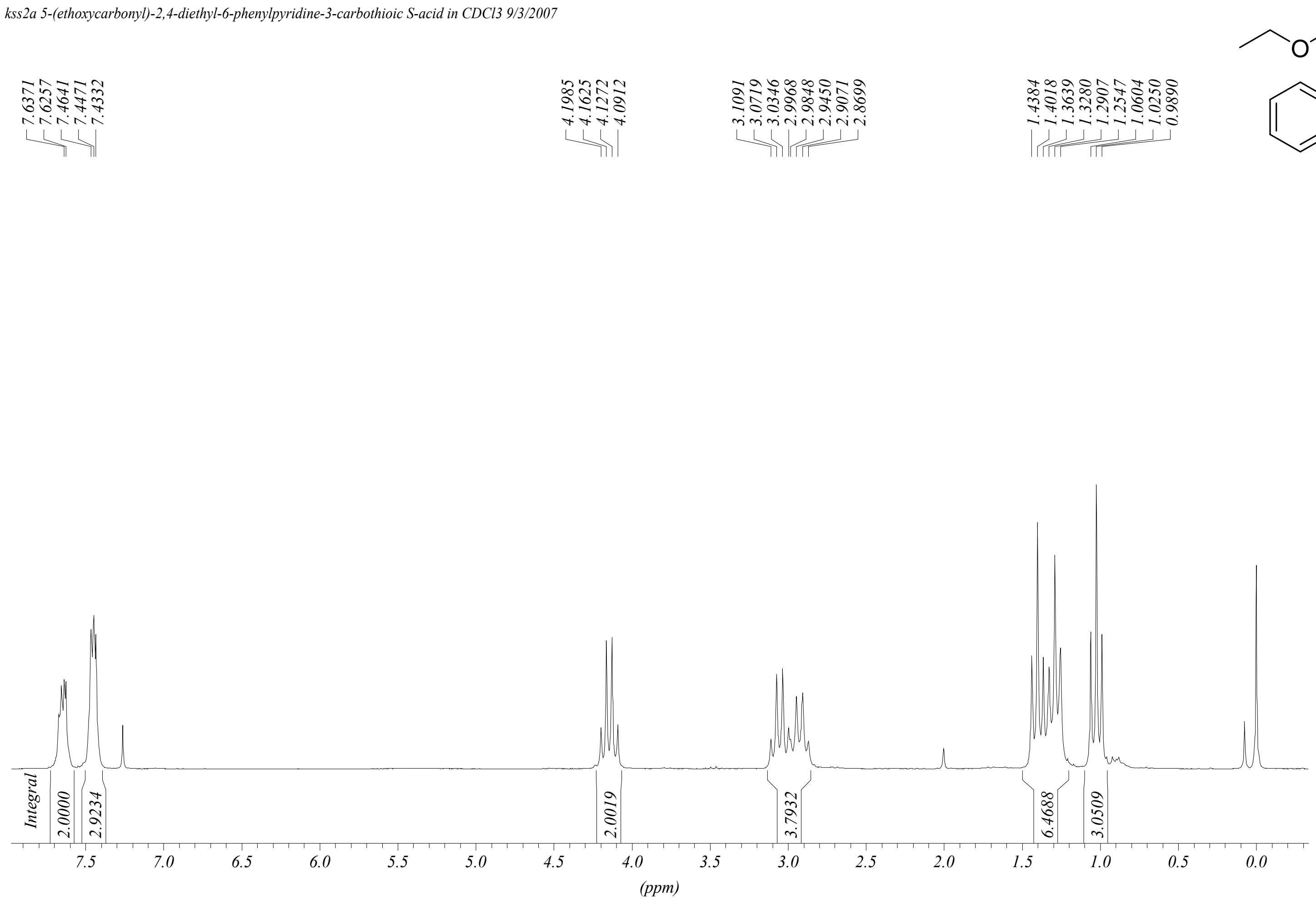

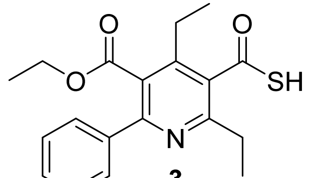

3

Figure S6. ^{13}C-NMR spectrum compound **3**.

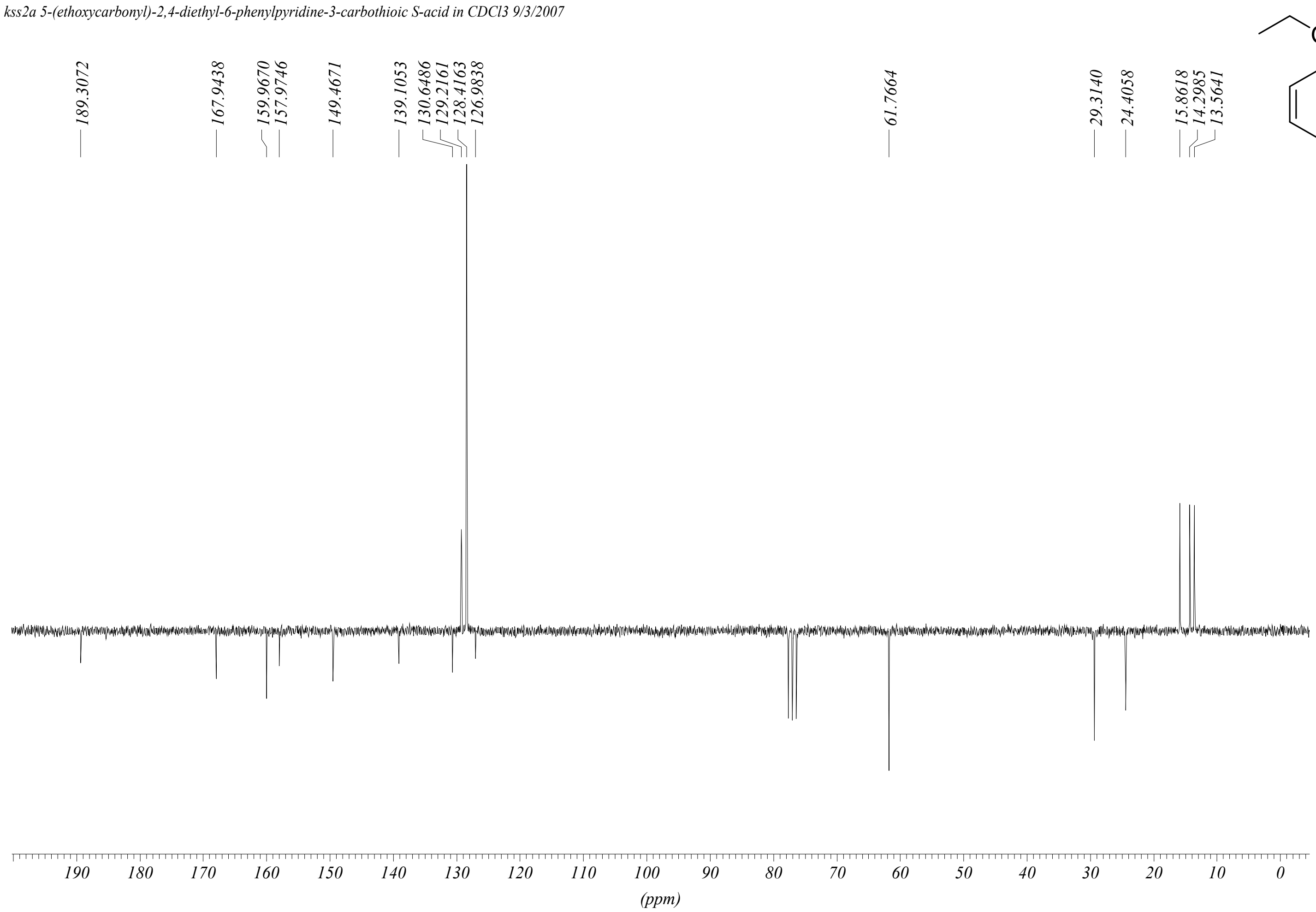

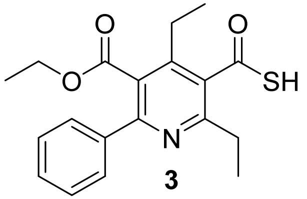

3

Figure S7. IR spectrum compound **3**.

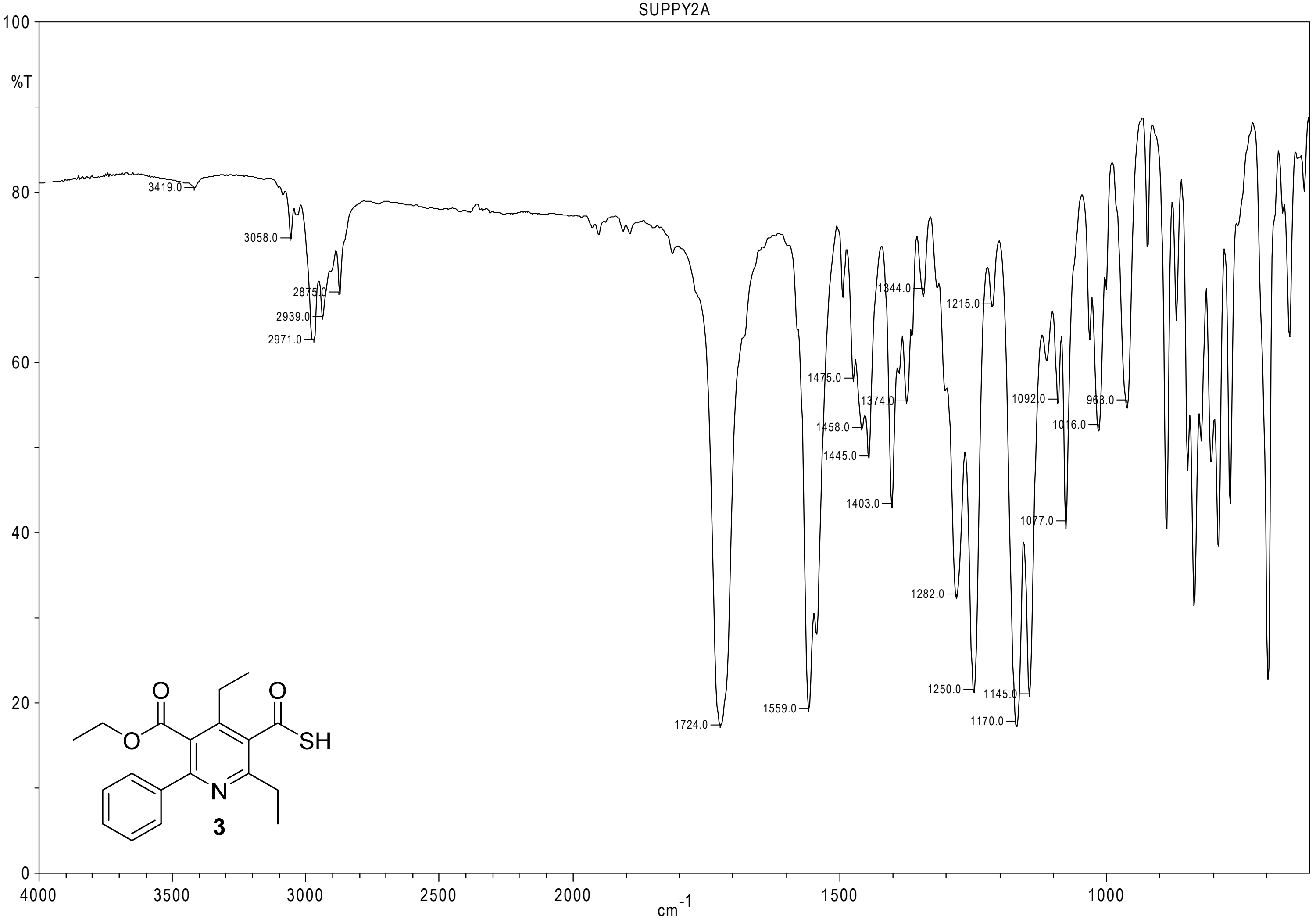

Figure S8. Mass spectrum compound **3**.

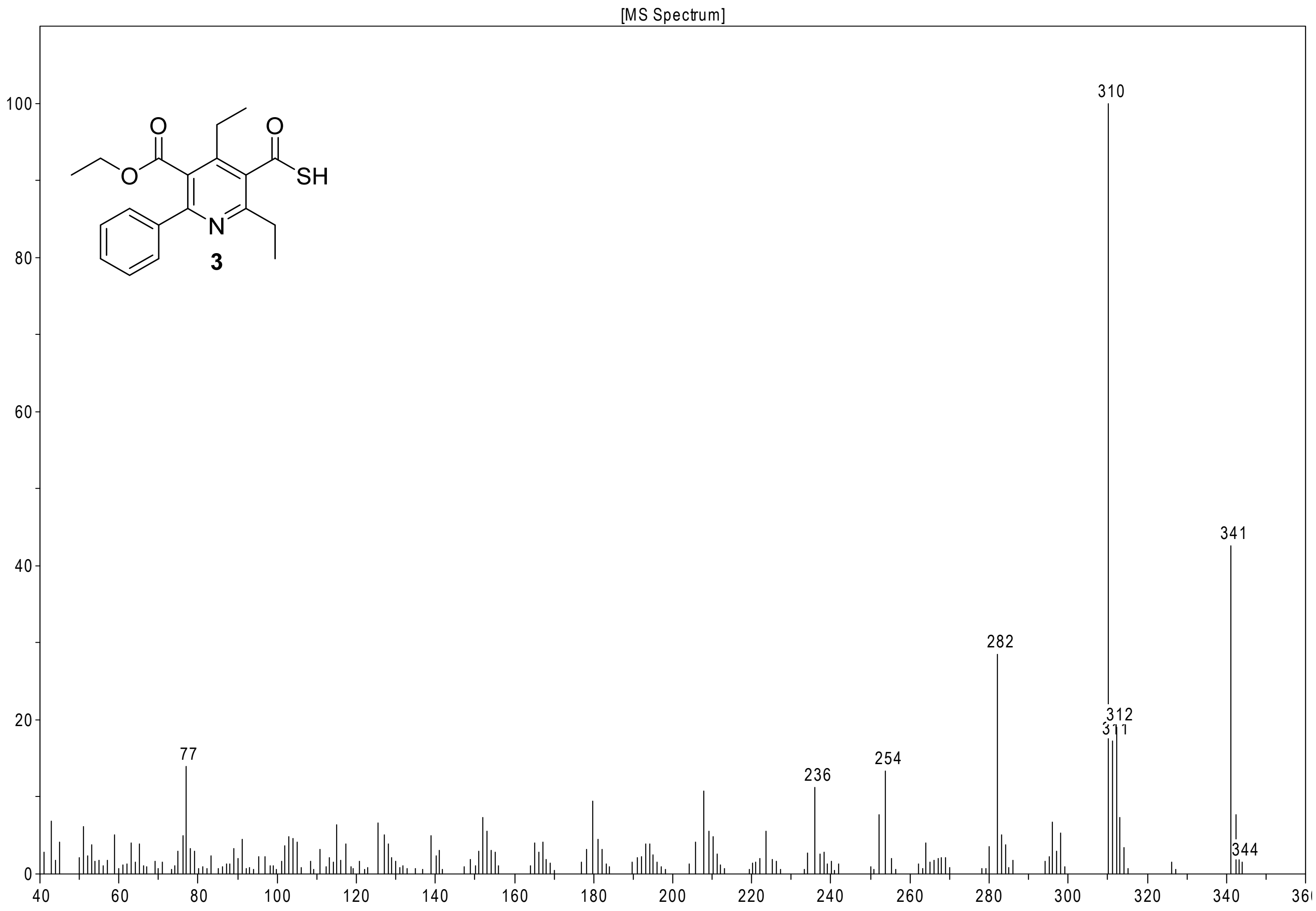

Figure S9. ^{1}H-NMR spectrum compound **4**.

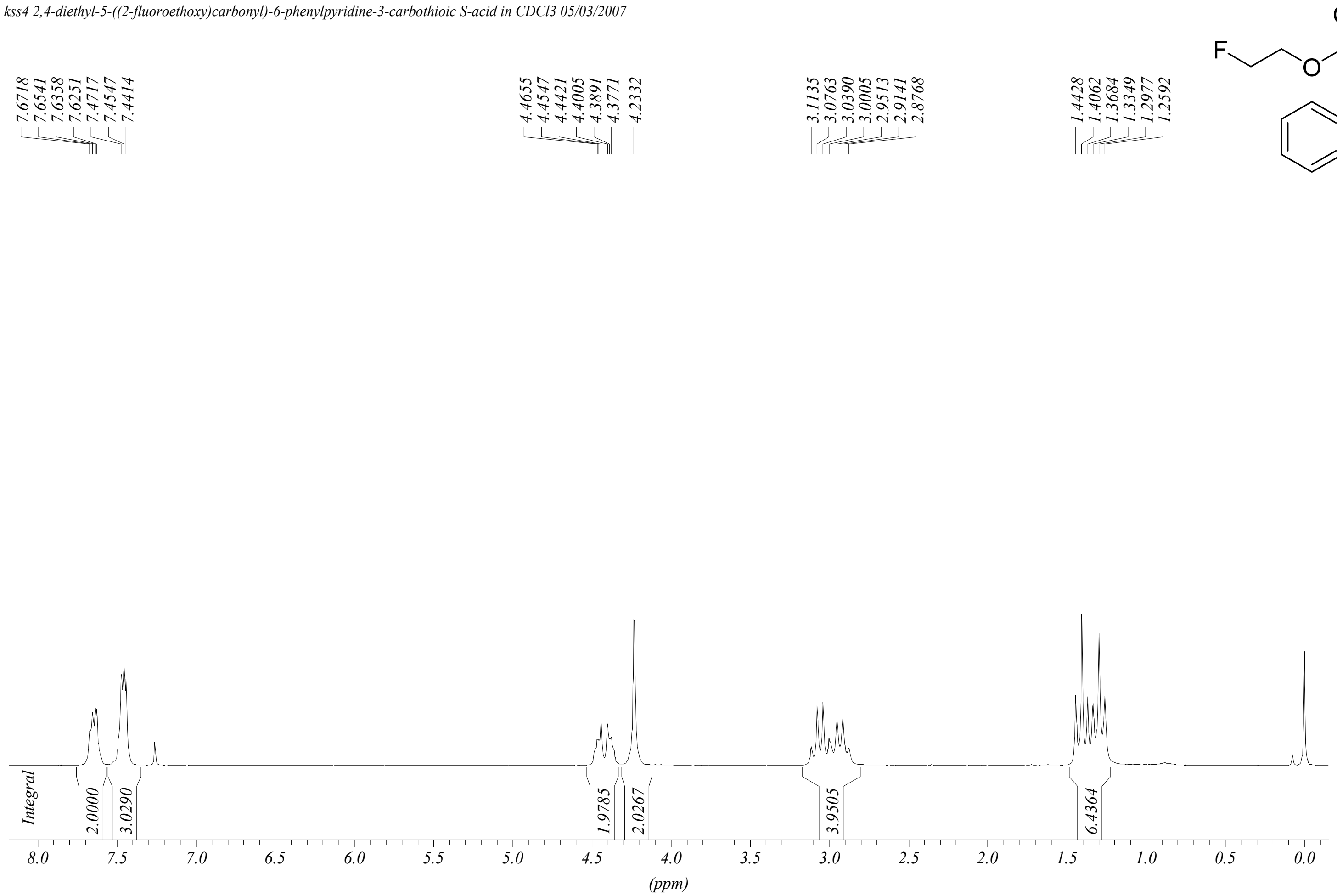

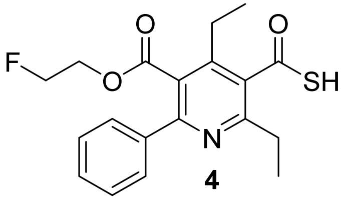

Figure S10. ^{13}C-NMR spectrum compound **4**.

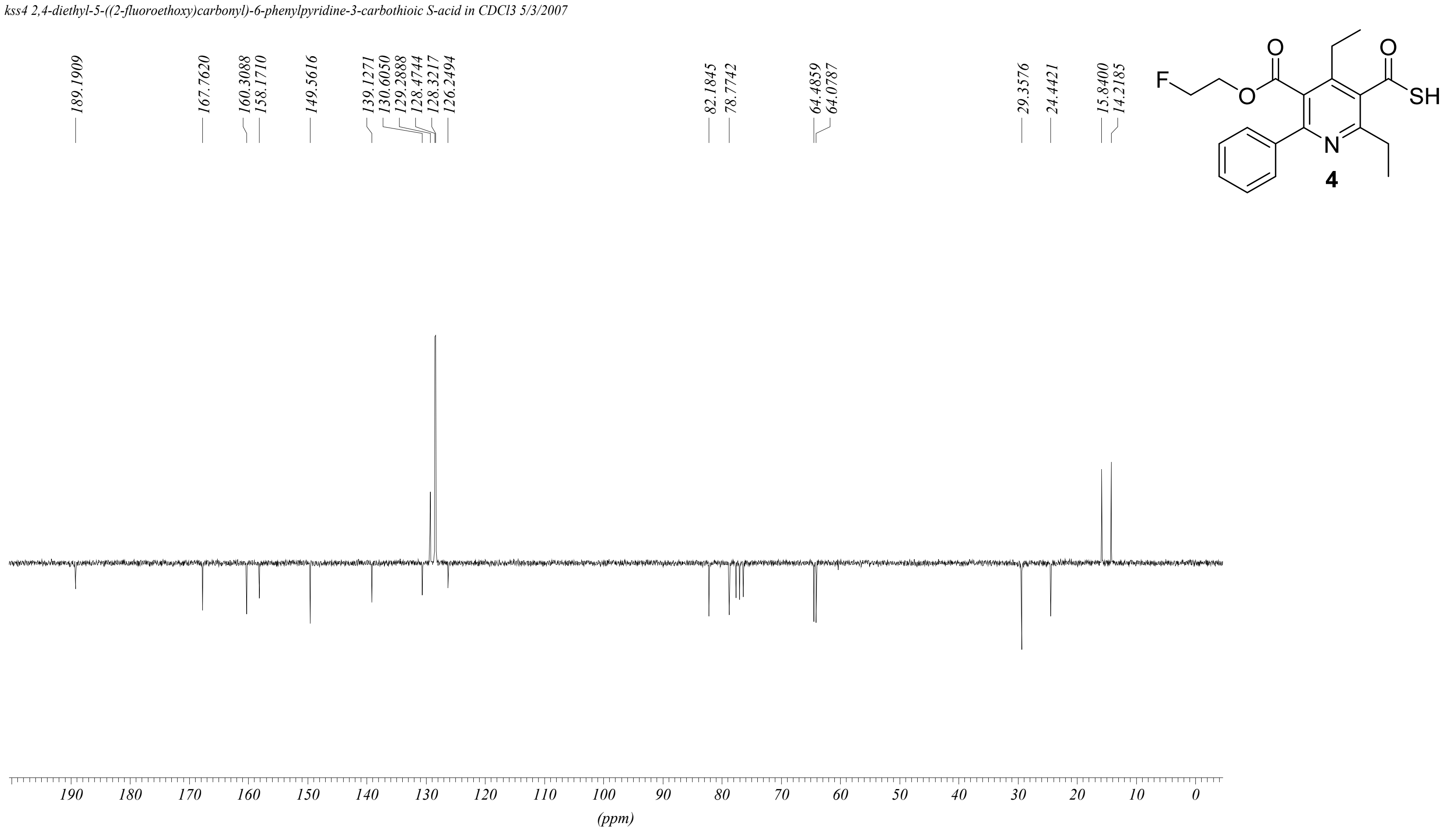

Figure S11. IR spectrum compound **4**.

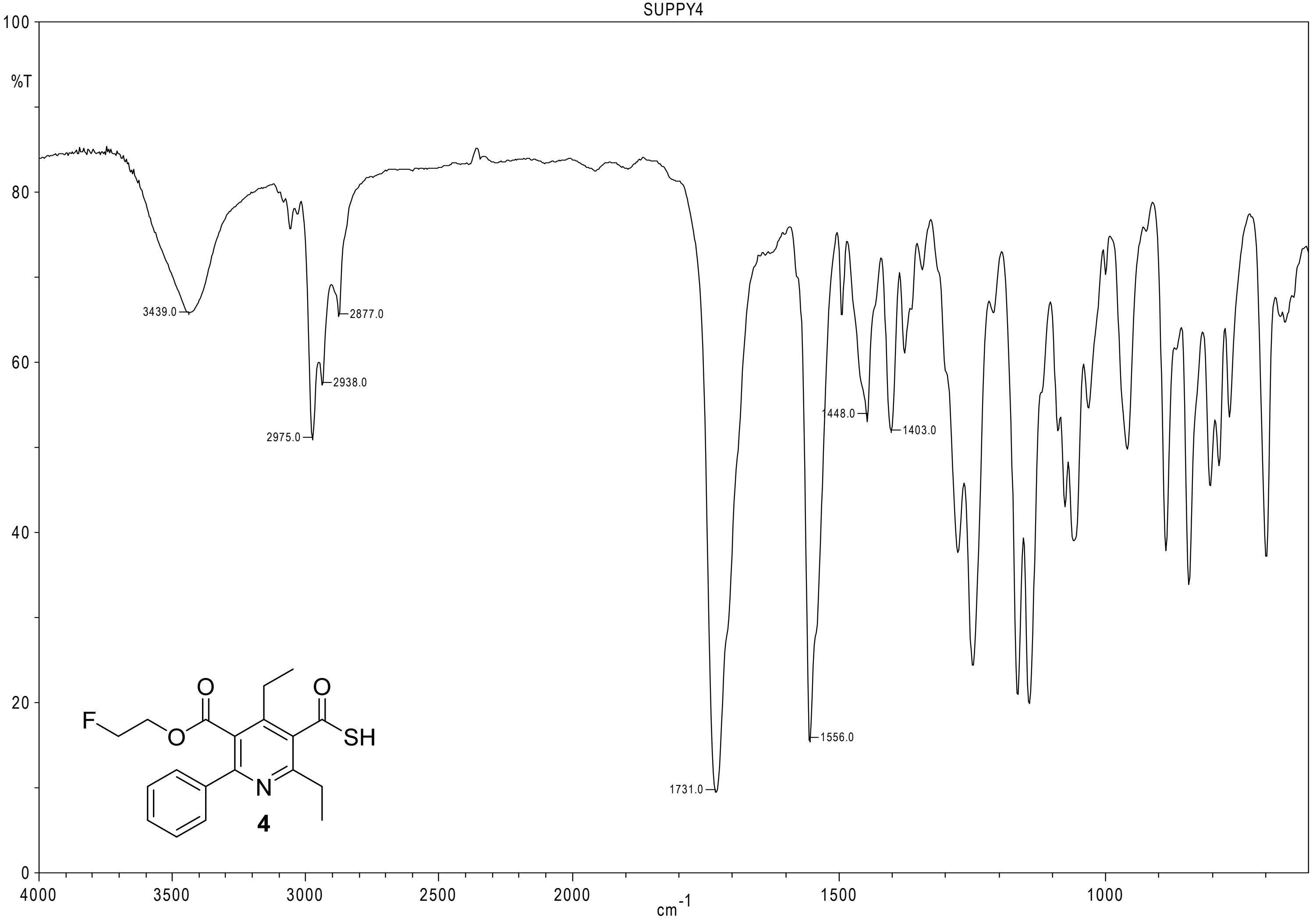

Figure S12. Mass spectrum compound **4**.

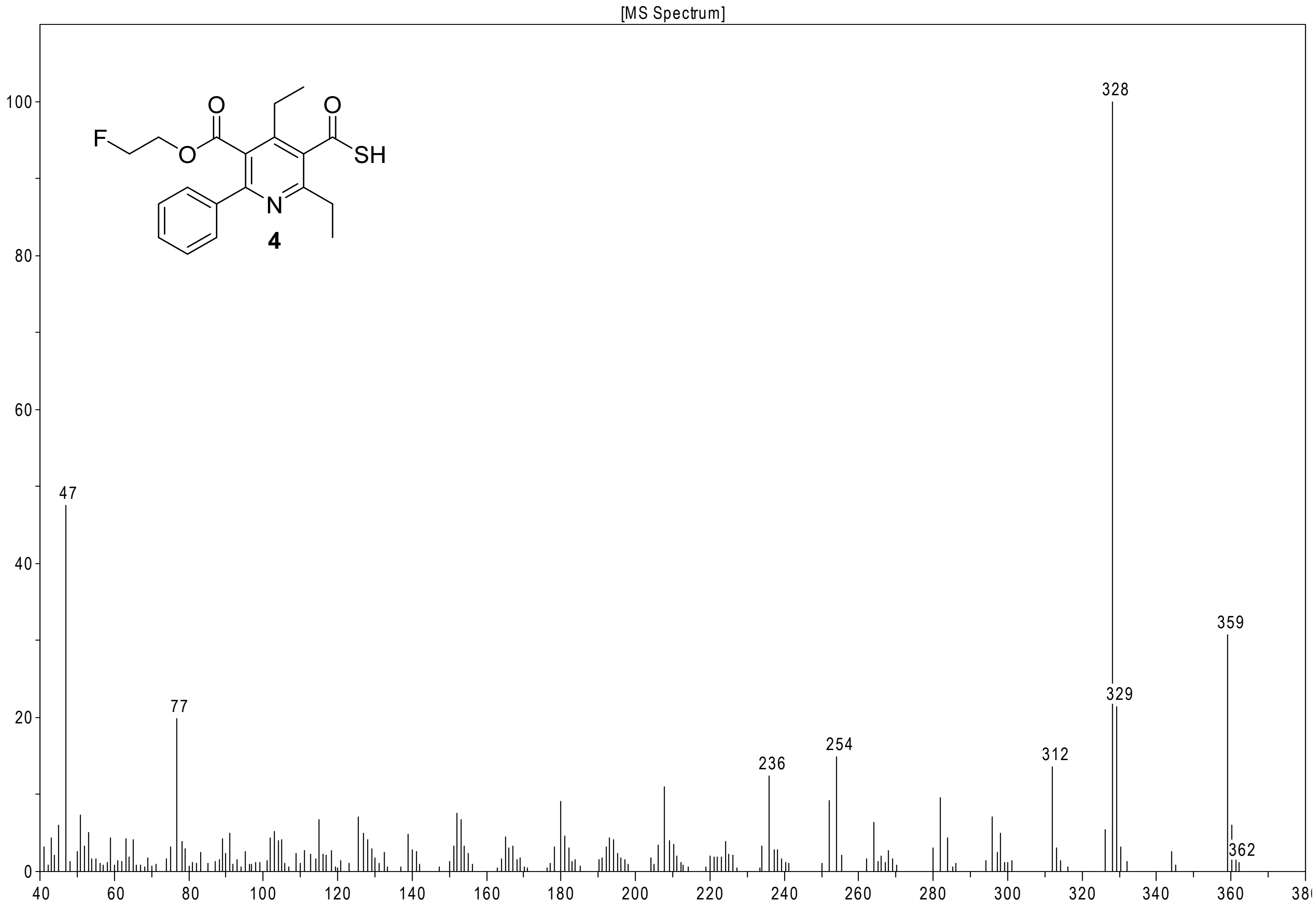

Figure S13. ^{1}H-NMR spectrum compound **5**.

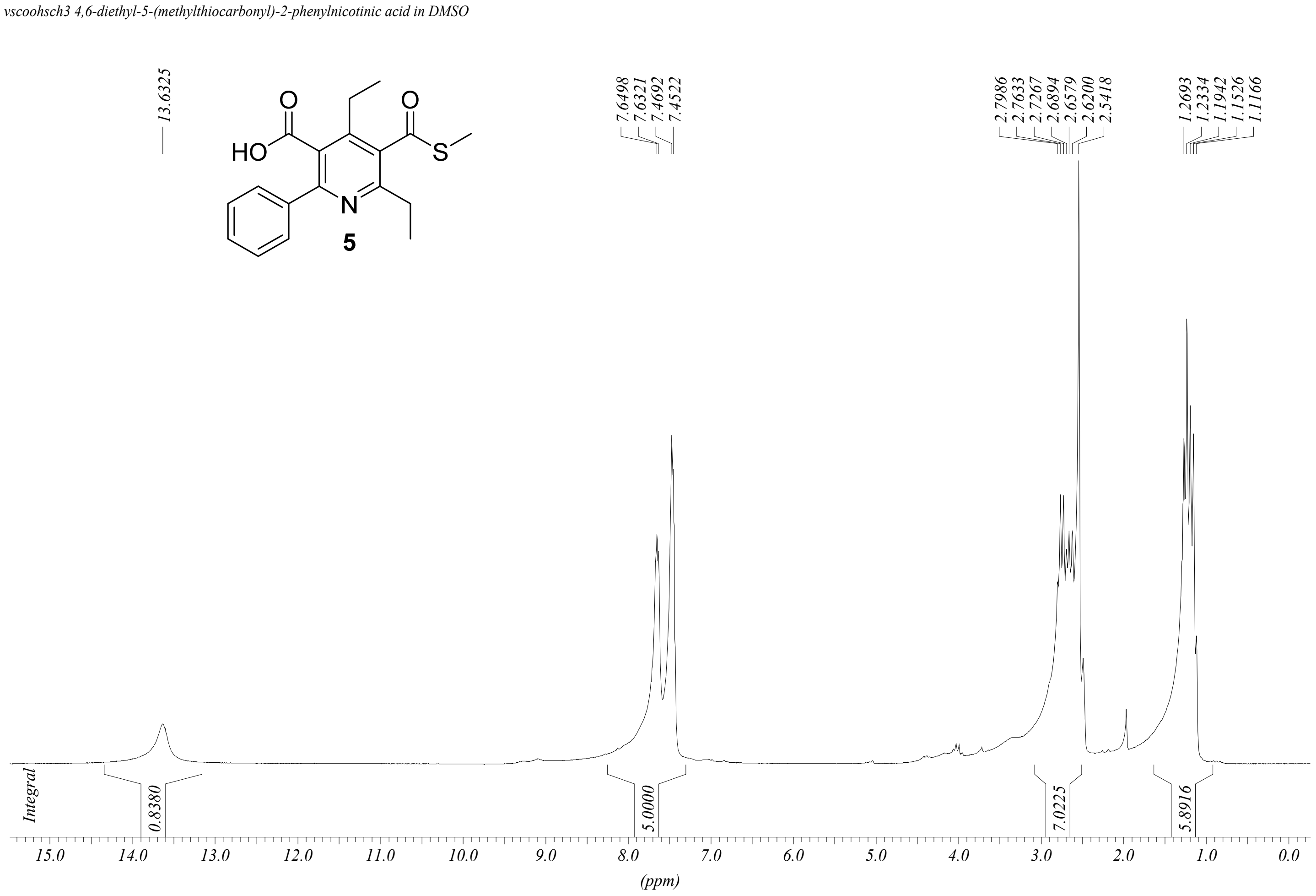

Figure S14. ^{13}C-NMR spectrum compound **5**.

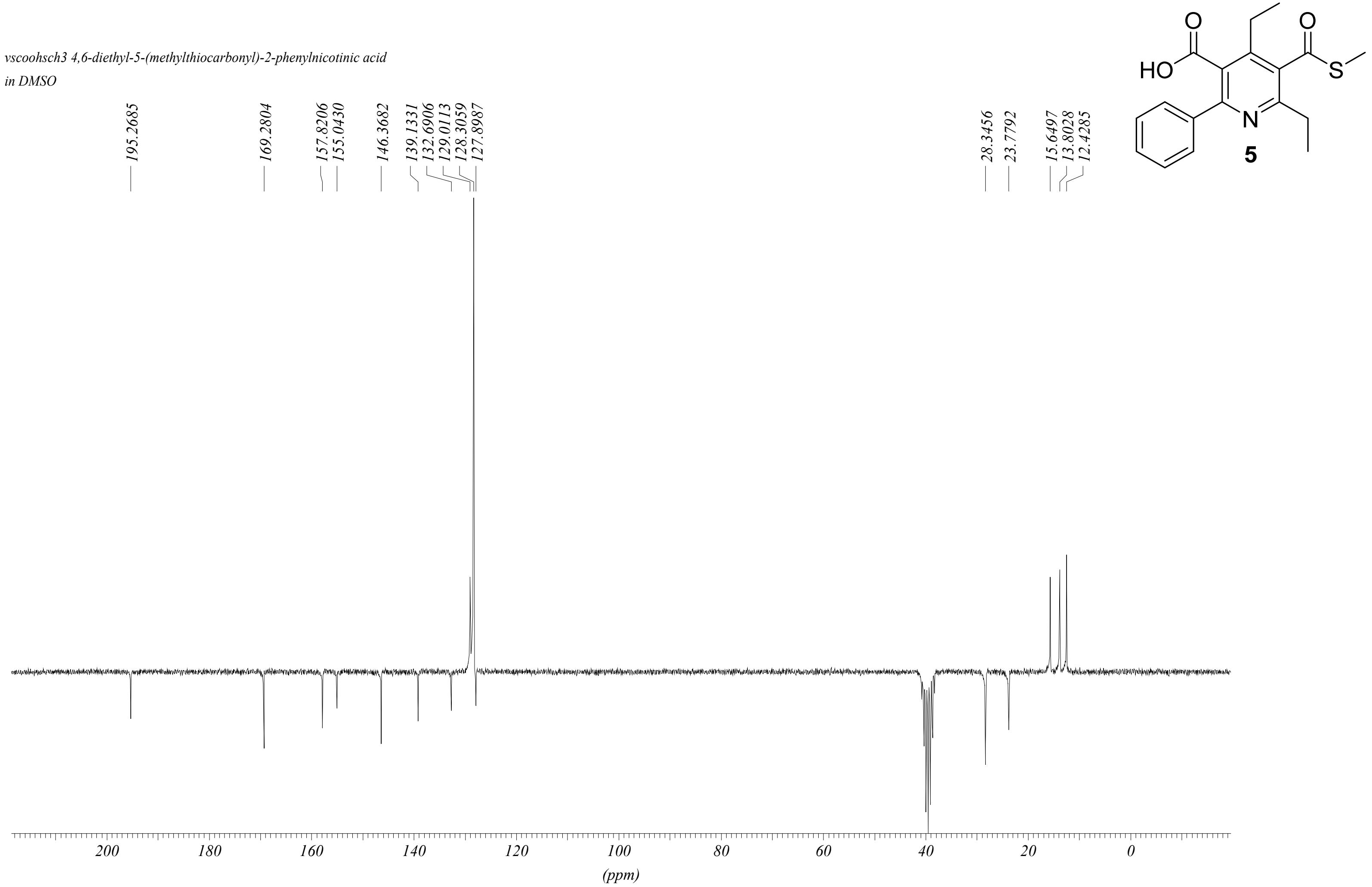

Figure S15. IR spectrum compound **5**.

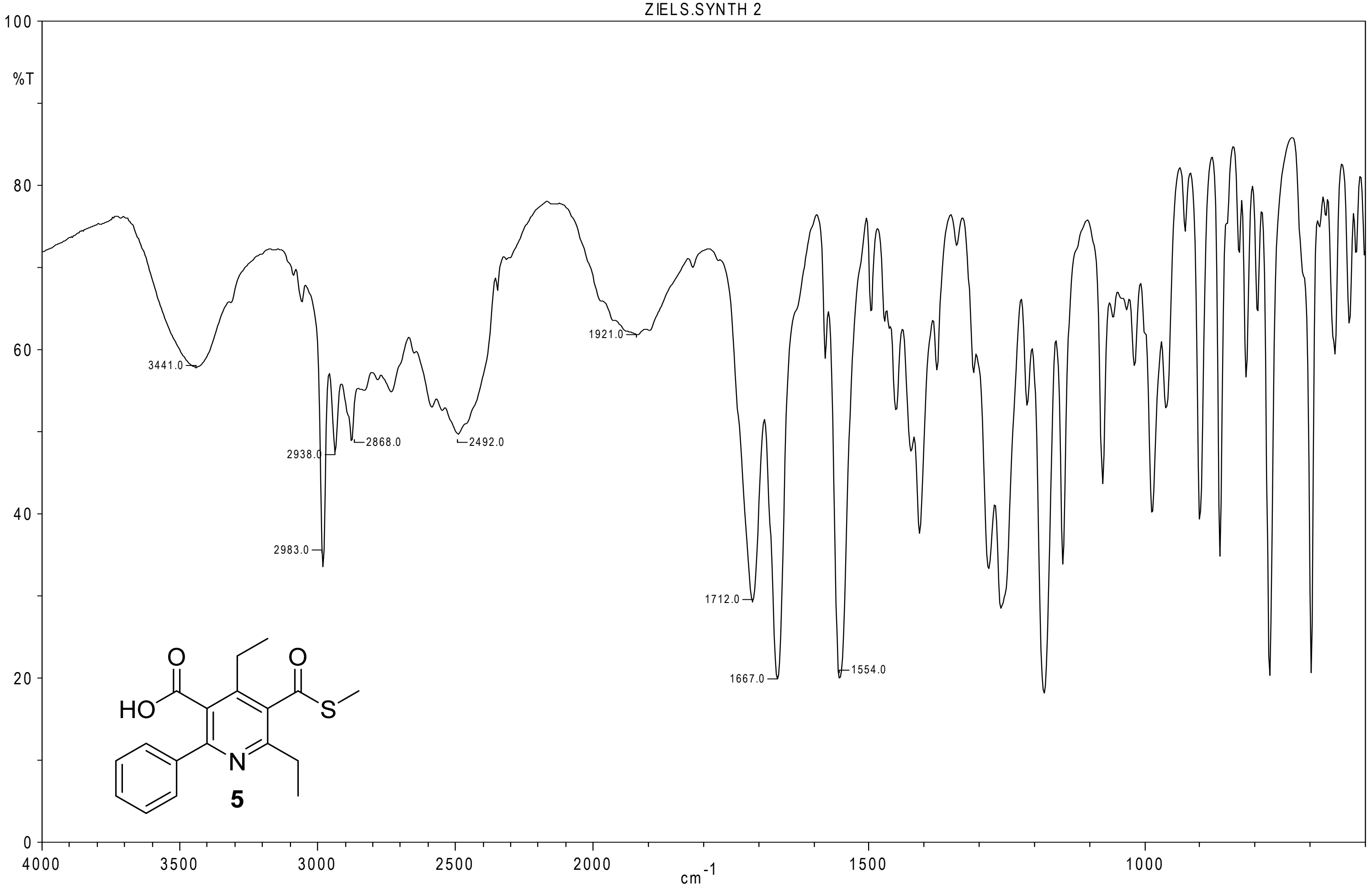

Figure S16. Mass spectrum compound **5**.

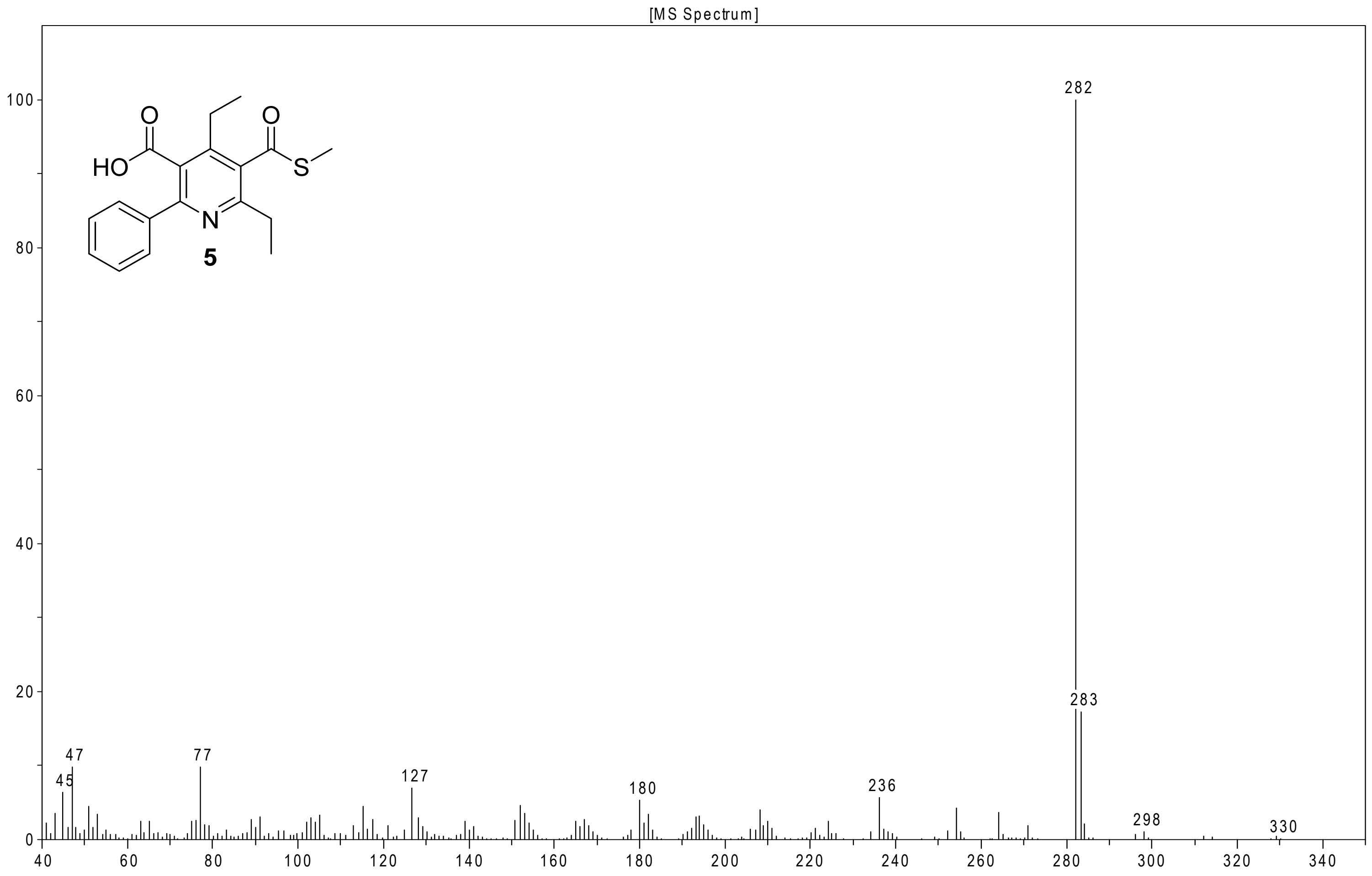

Figure S17. ^{1}H-NMR spectrum compound **6**.

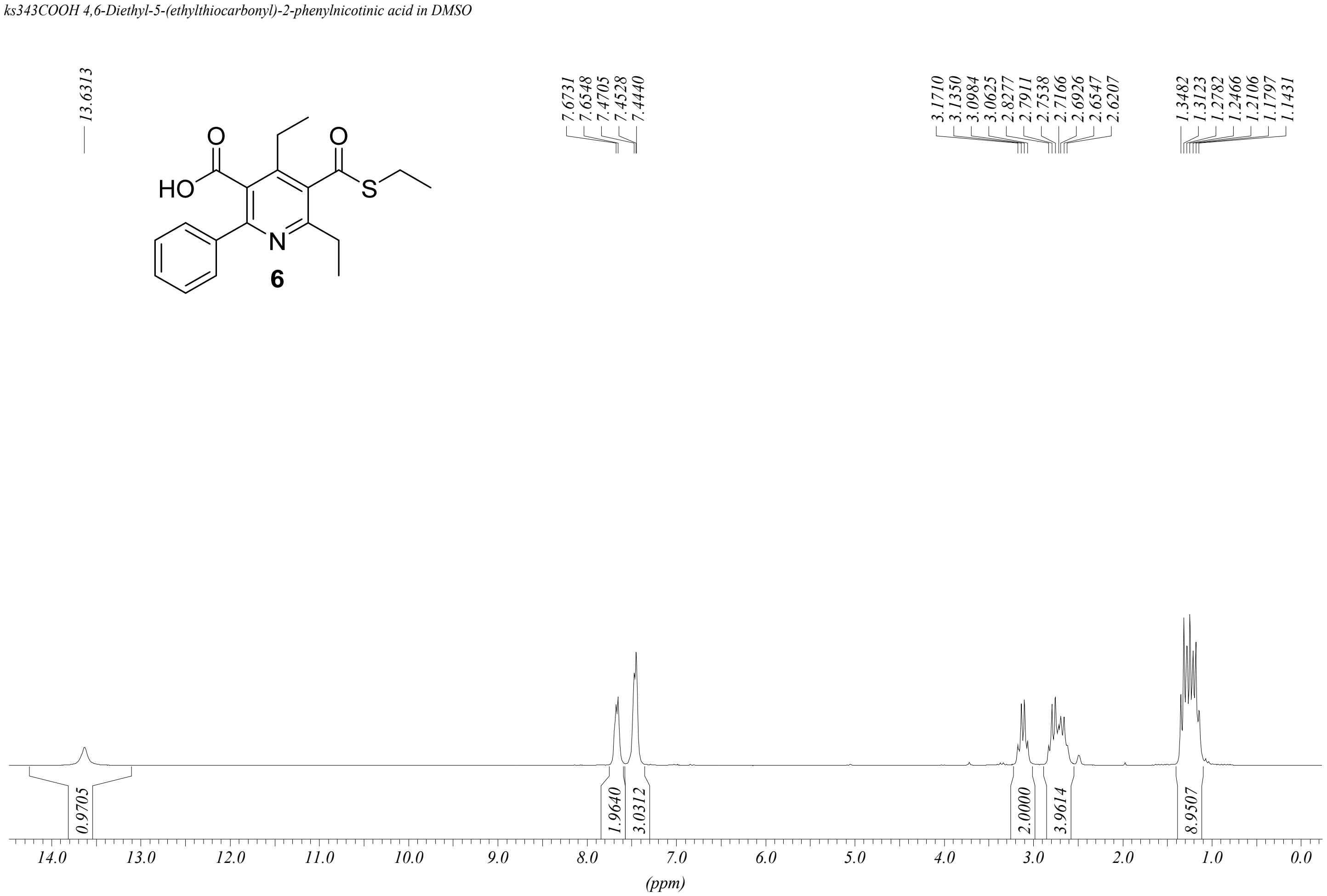

Figure S18. ^{13}C-NMR spectrum compound **6**.

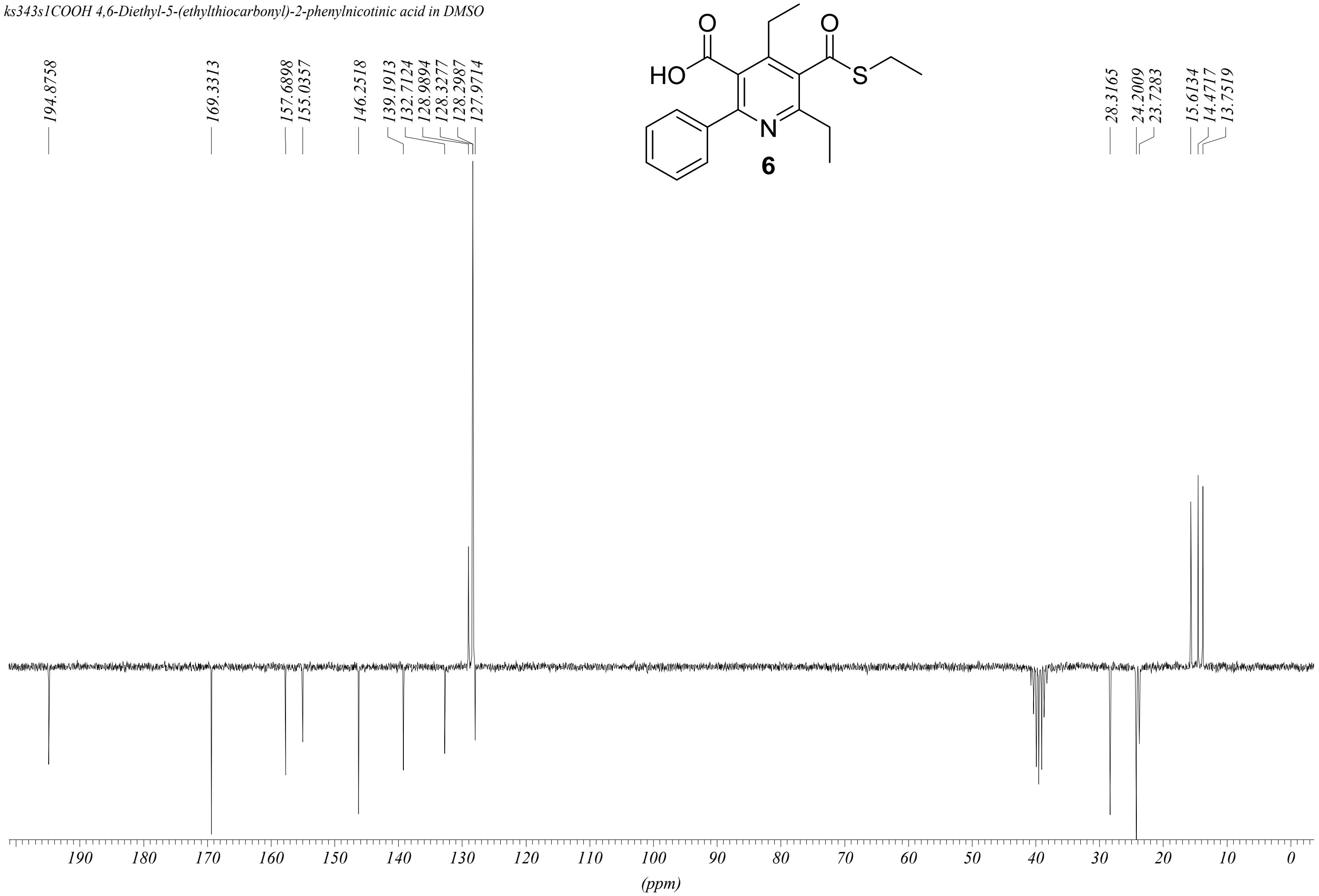

Figure S19. IR spectrum compound **6**.

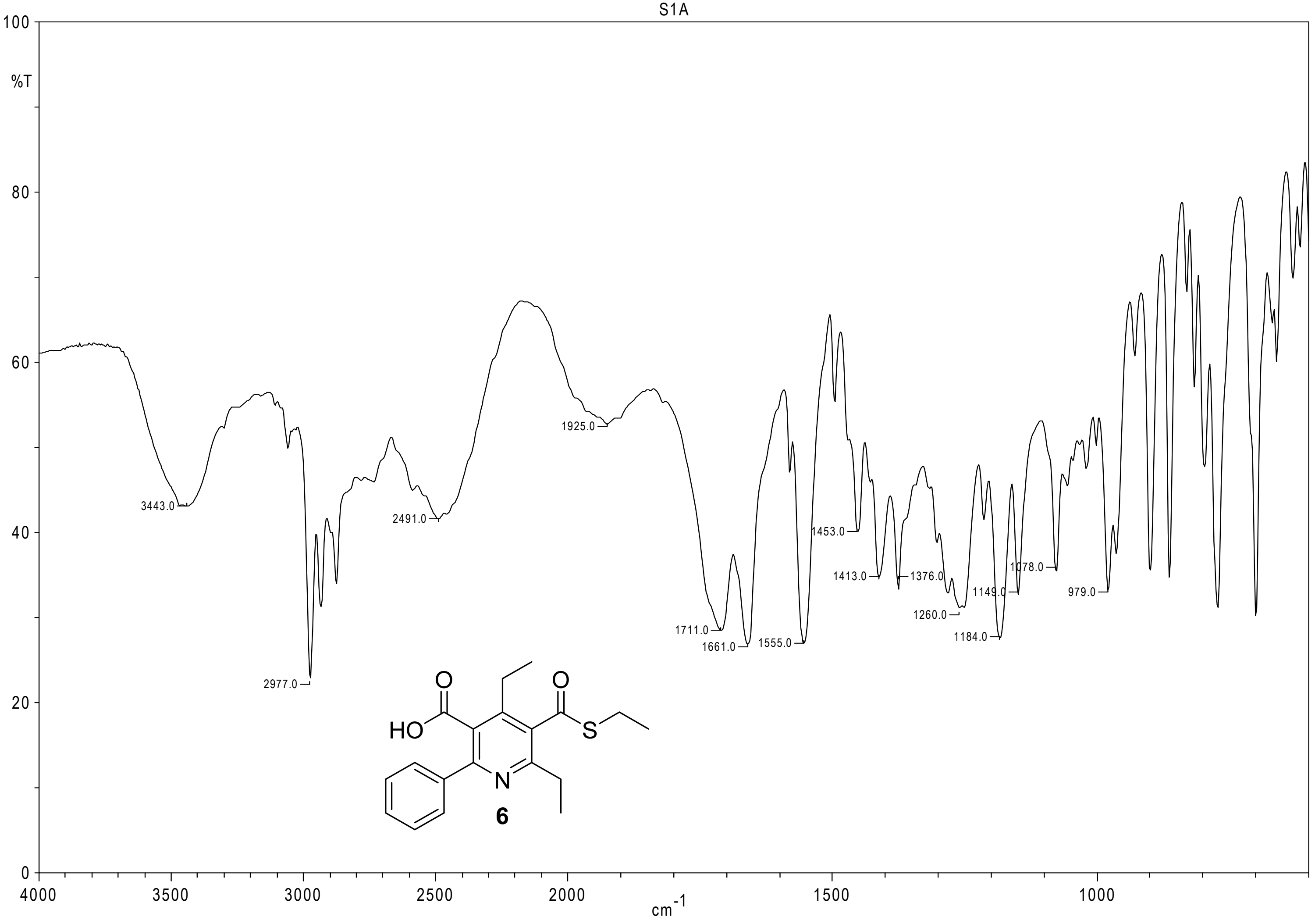

Figure S20. Mass spectrum compound **6**.

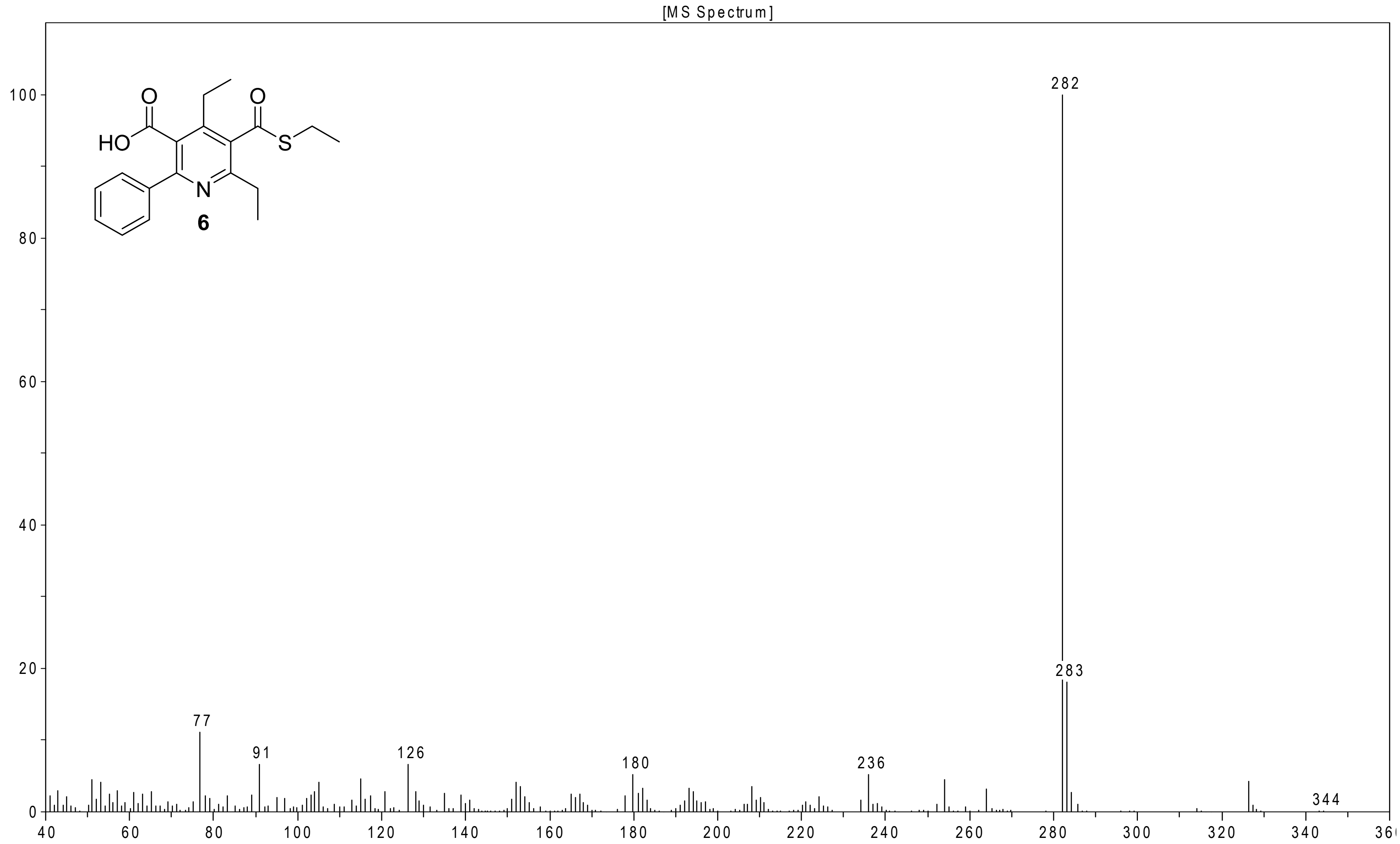

Figure S21. ^{1}H-NMR spectrum compound **7**.

kss5 4,6-diethyl-5-((2-fluoroethylthio)carbonyl)-2-phenylnicotinic acid in DMSO 31/5/2007

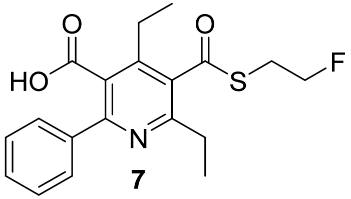

7

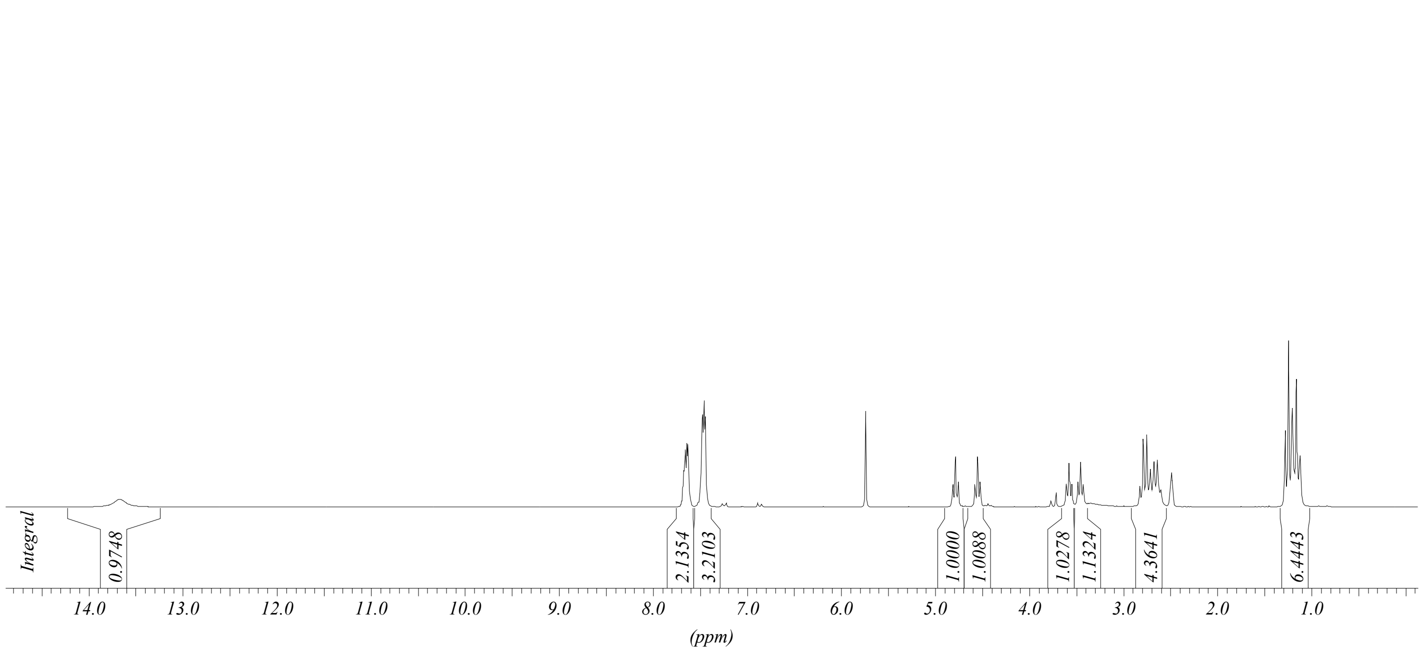

Figure S22. ^{13}C-NMR spectrum compound **7**.

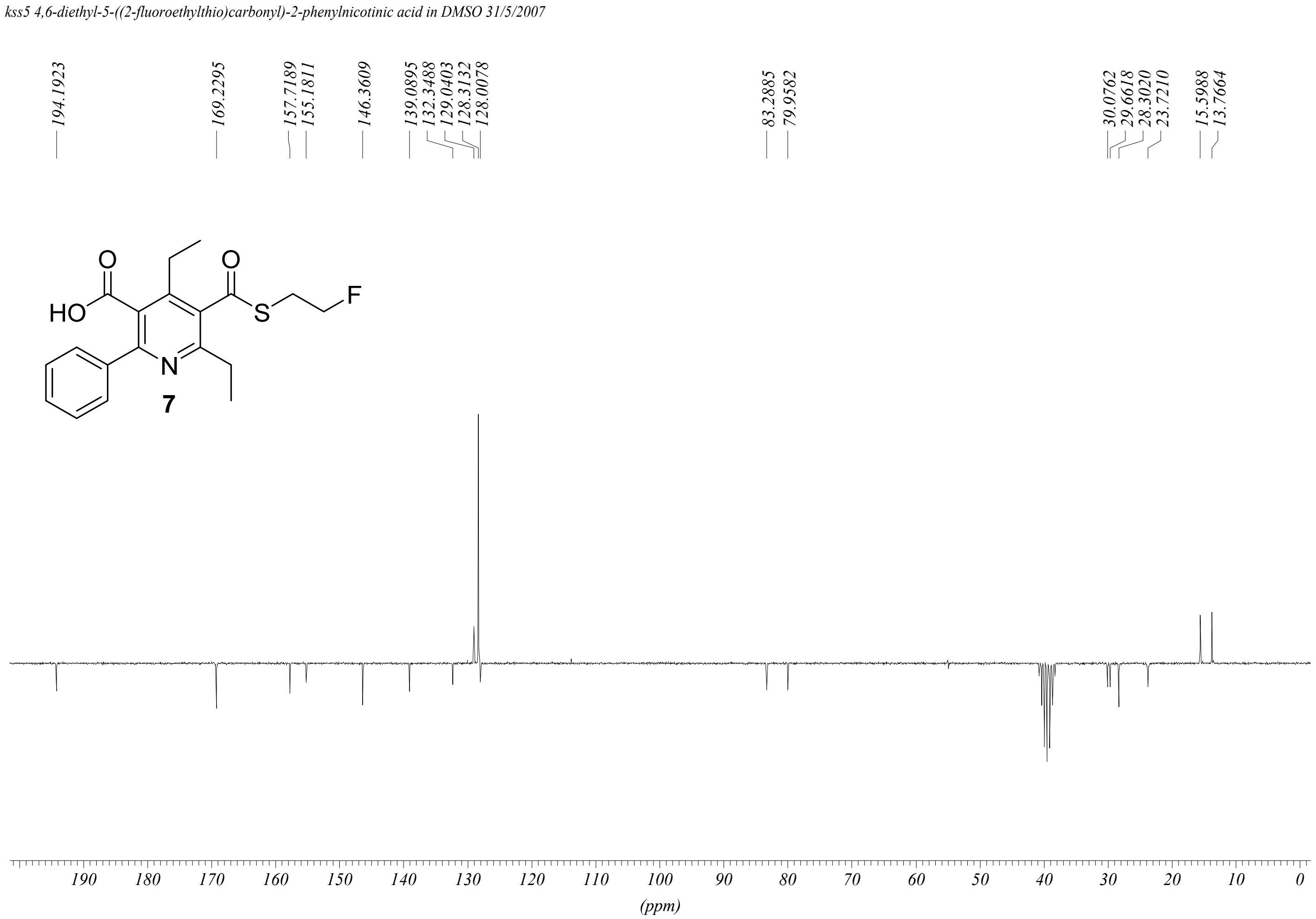

Figure S23. IR spectrum compound **7**.

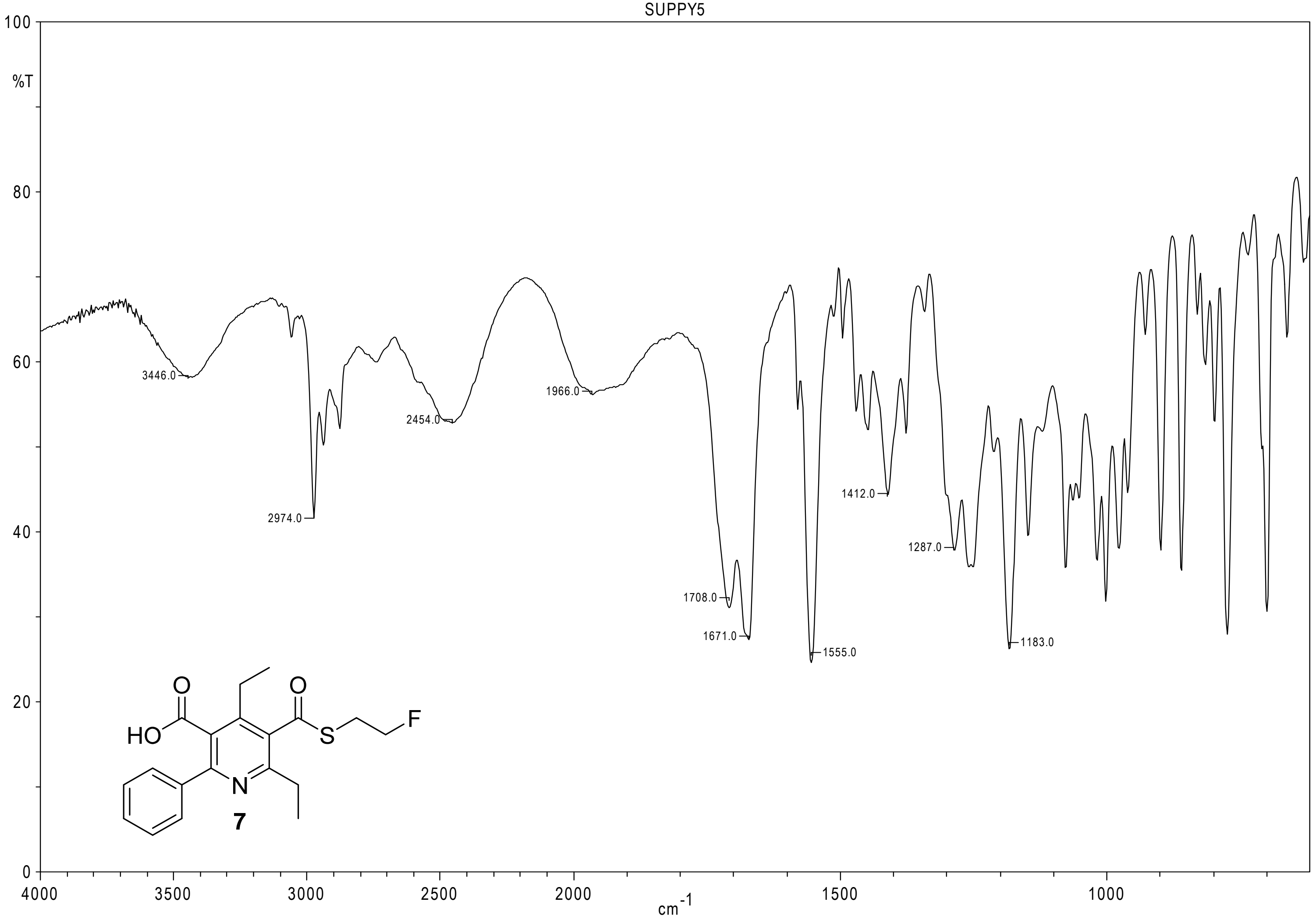

Figure S24. Mass spectrum compound **7**.

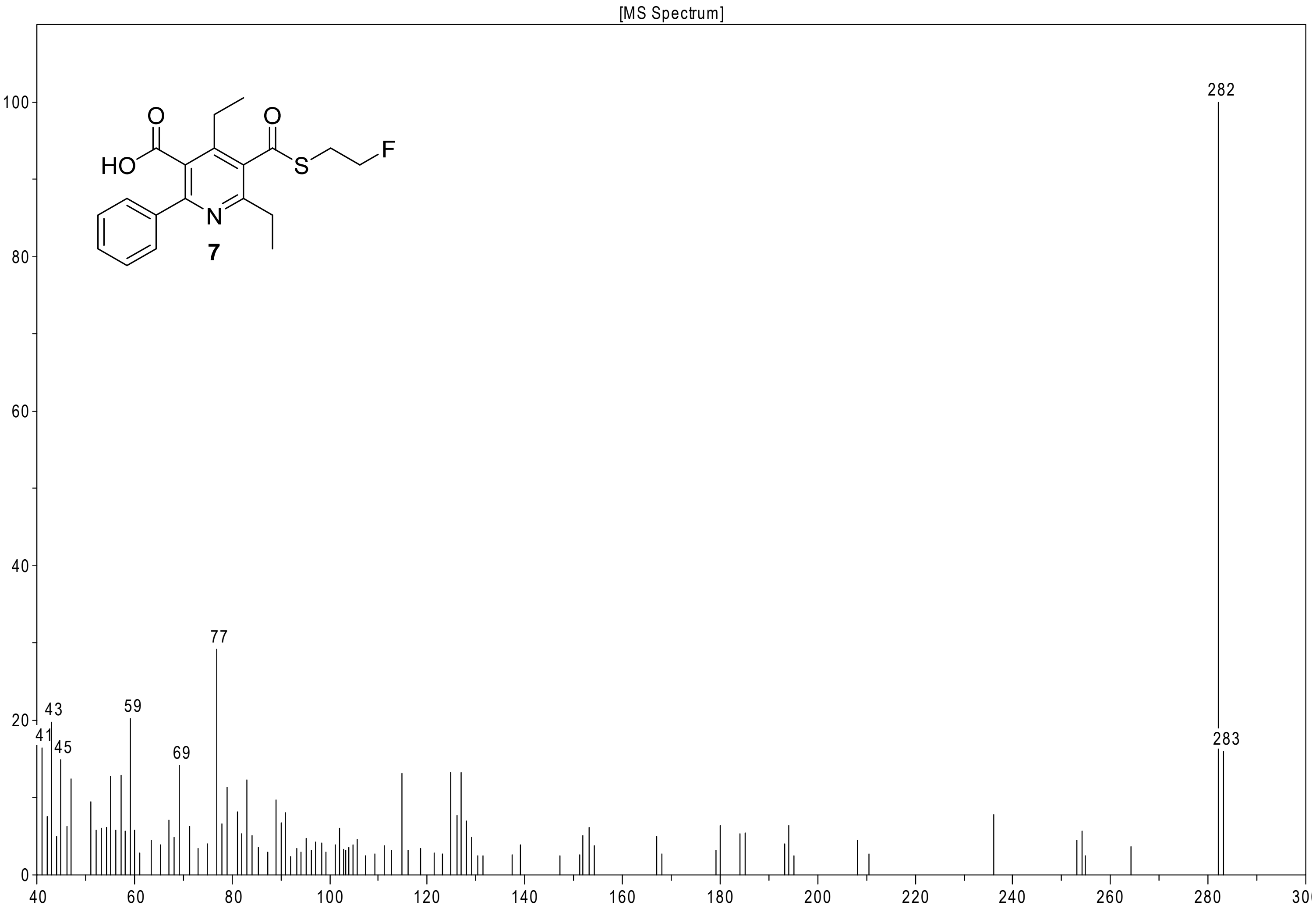

Figure S25. ^{1}H-NMR spectrum compound **8**.

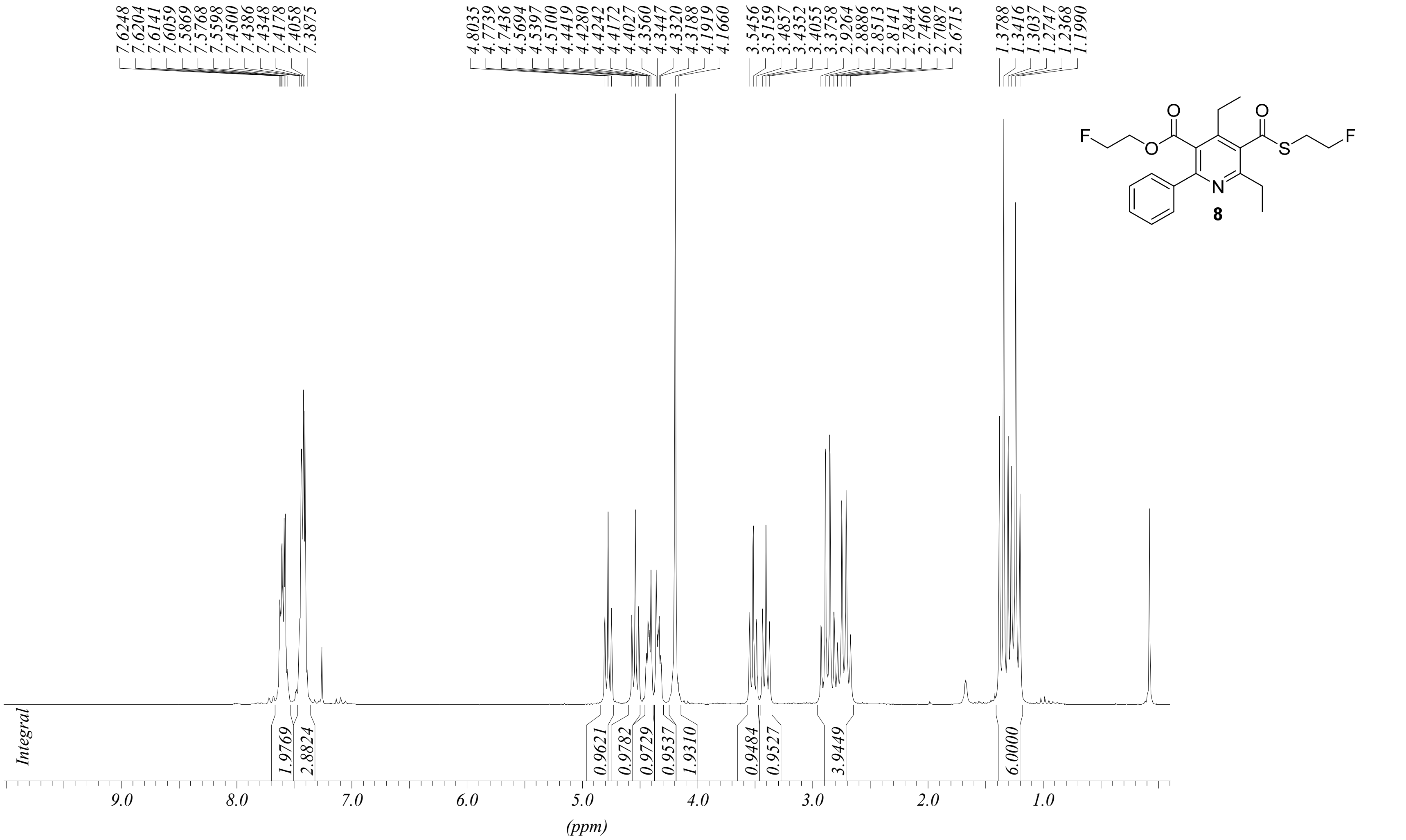

Figure S26. ^{13}C-NMR spectrum compound **8**.

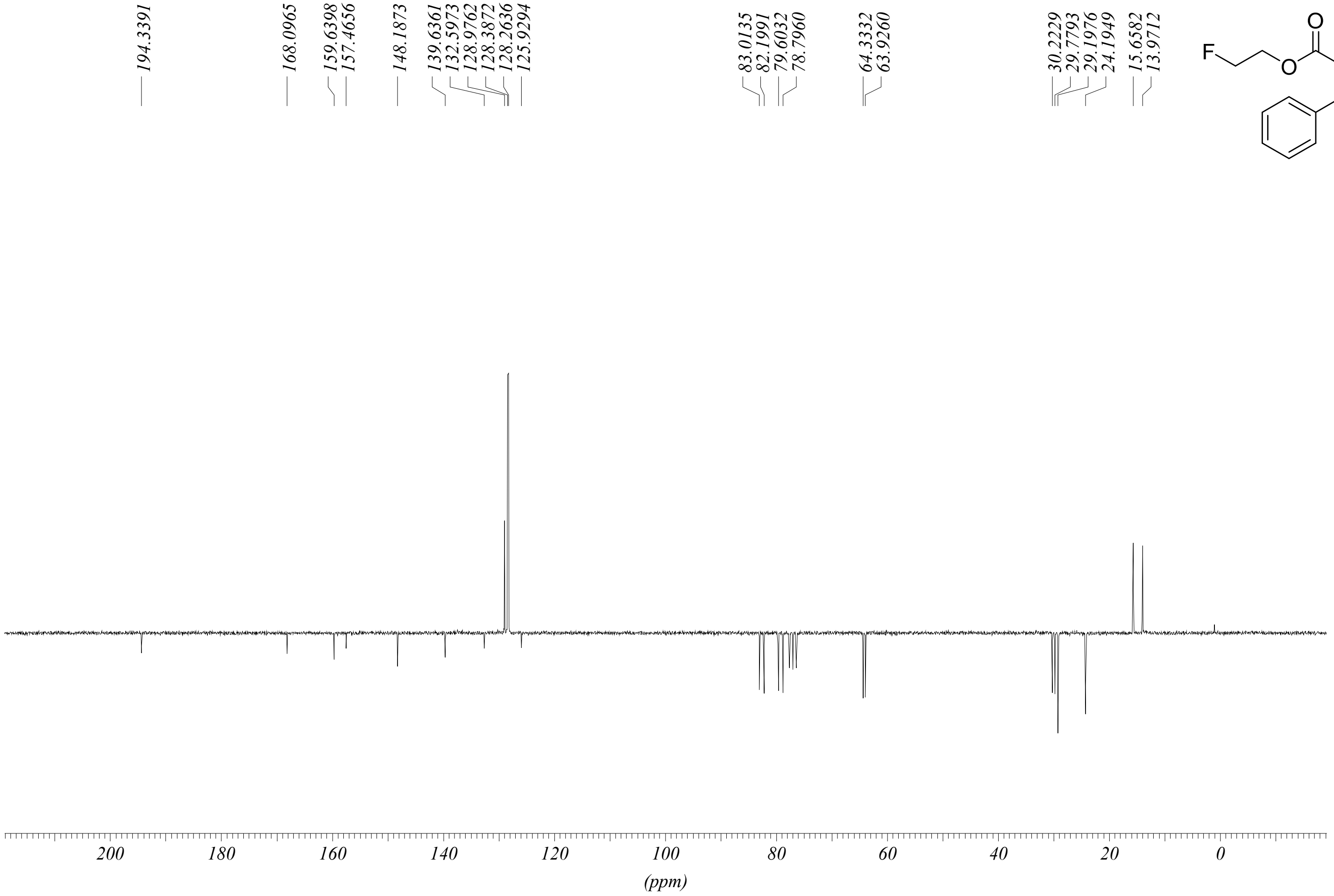

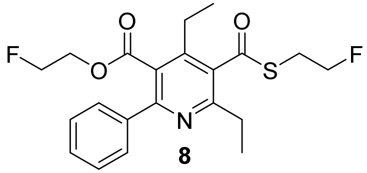

8

Figure S27. IR spectrum compound **8**.

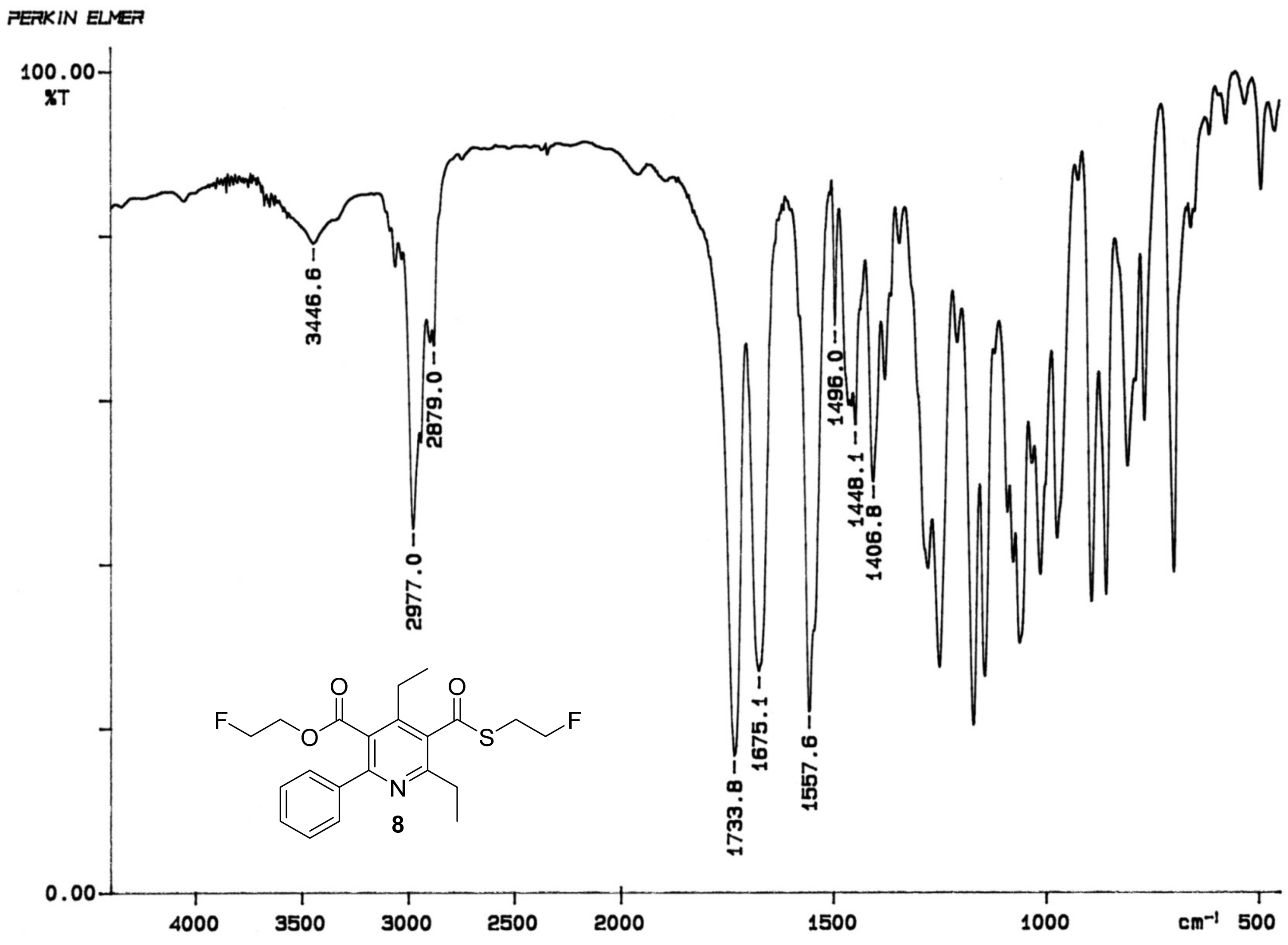

Figure S28. Mass spectrum compound **8**.

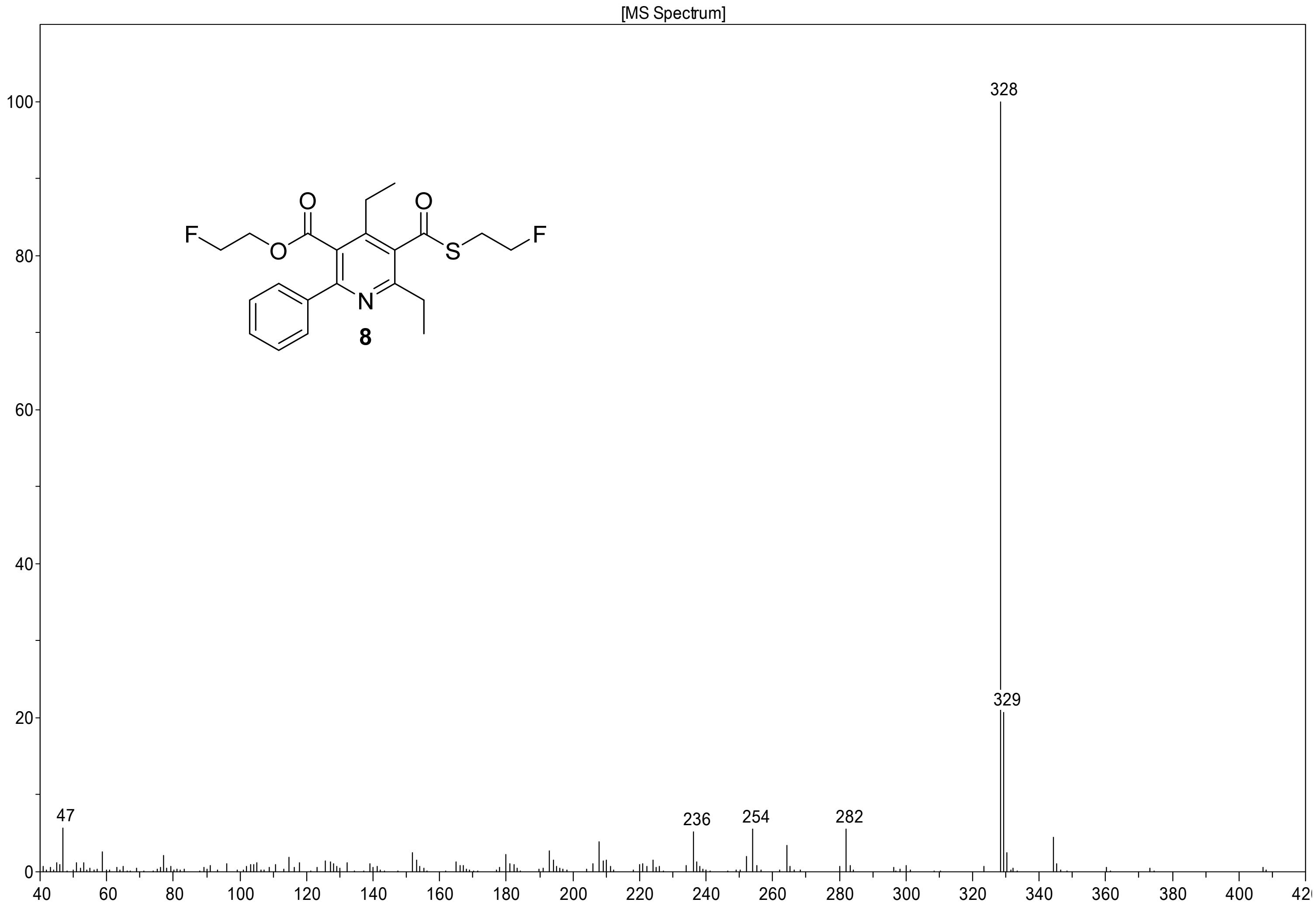

Figure S29. Mass spectrum compound **8**

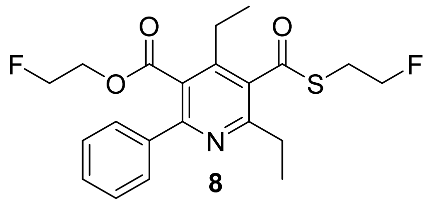


```
SPEC:  000000000000000000000000000000     16-Nov-92  Elapse:  00:25.6       1
Samp:  TN407                                         Start :  14:29:52      1
Comm:  4kV  0.3uA    MeOH/ACN
Mode:  ESI +VE +LMR  BSCAN (EXP) UP LR NRM           Study :  ESI
Oper:  phu           Client:  Nagel/Pharm            Inlet :
Base:  408.2                Inten :  851245          Masses:  100 > 1298
Norm:  408.2                RIC   :  1632384         #peaks:  1241
Peak:  1000.00  mmu
Data:  AVER : Scans 1-10  from /usr/users/finnigan/data/36074_1.dat
```

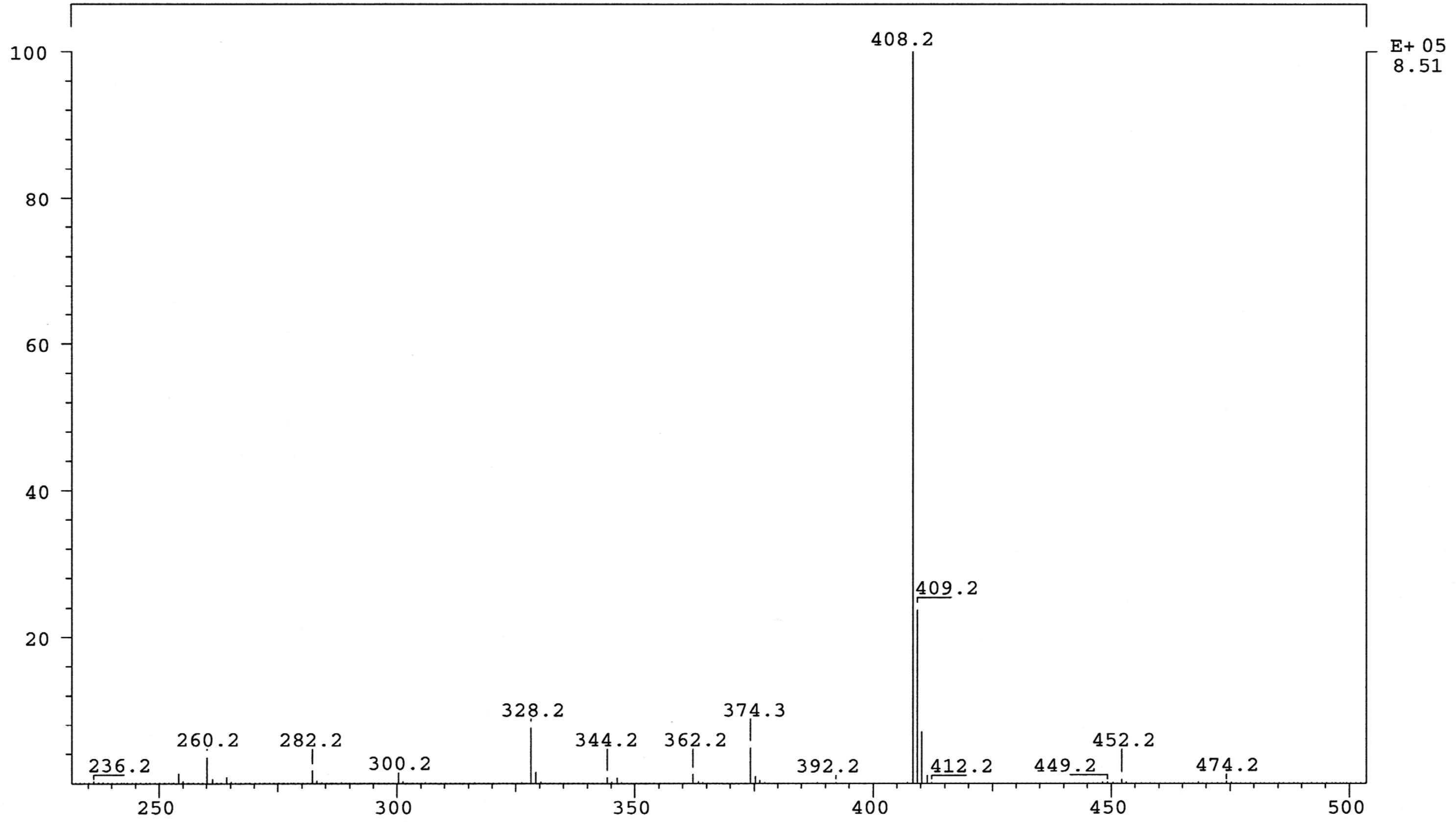

Supplement: Supplementary file 1 [file molecules-19-04076-s001.pdf]
